# Supplementary material for: No such thing as bad publicity? A quantitative content analysis of print media representations of primary care out-of-hours services
Source: BMJ Open. 2019 Mar 25;9(3):e023192. doi: 10.1136/bmjopen-2018-023192 (PMC6475237; doi:10.1136/bmjopen-2018-023192)
Supplement: Supplementary data [file bmjopen-2018-023192supp001.pdf]

Appendix 1. Overview of main UK and Scottish daily and Sunday newspapers selected by publication location, genre, circulation, readership and political ideology, October 205 – September 2016. (From: National Readership Survey, http://www.nrs.co.uk/downloads/pdf/newspapers_201611.pdf)

Newspaper	Estimated weekly readership (Number)	% Readership Female	% Readership in Social Group C2DE	% Readership Aged 35 +	Political Ideology
**UK**					
**Quality**					
Daily Telegraph	1,183,000	47.4	17.8	89.6	Right
The Sunday Telegraph	1,113,000	46.9	15.5	89.2	Right
The Times	1,047,000	44.6	15.2	83.8	Centre-right
The Sunday Times	1,834,000	47.0	15.4	83.9	Centre-right
The Guardian	865,000	48.4	20.1	73.9	Centre-left
The Observer	764,000	45.8	13.2	80.9	Centre-left
**Middle-Market**					
Daily Mail	3,354,000	53.9	38.0	87.7	Right
The Mail on Sunday	3,608,000	51.5	39.2	87.0	Right
Daily Express	842,000	48.8	43.6	91.8	Right
Sunday Express	826,000	47.5	40.4	88.0	Right
**Tabloid**					
The Sun*	4,188,000	42.7	68.3	75.5	Right
The Sun on Sunday*	3,640,000	44.6	65.3	72.4	Right
Daily Mirror	1,818,000	46.1	65.0	83.7	Left
Sunday Mirror	1,804,000	49.8	61.5	79.0	Left
Daily Star	800,000	34.5	75.1	74.8	Neutral
Daily Star Sunday	584,000	40.8	74.5	71.2	Neutral

**Scotland**					
**Quality**					
The Herald	109,000	33.0	26.6	87.2	Neutral
Sunday Herald	109,000	46.8	30.3	73.4	Neutral
The Scotsman	73,000	42.5	21.9	87.7	Neutral
Scotland on Sunday	72,000	51.4	18.1	84.7	Neutral
**Tabloid**					
Daily Record	484,000	48.3	69.4	89.1	Left
Sunday Mail	558,000	50.7	67.0	89.1	Left

*Not included in this analysis, as The Sun is not included in the Nexis database; included here for comparison.
