# Supplementary material for: No such thing as bad publicity? A quantitative content analysis of print media representations of primary care out-of-hours services
Source: BMJ Open. 2019 Mar 25;9(3):e023192. doi: 10.1136/bmjopen-2018-023192 (PMC6475237; doi:10.1136/bmjopen-2018-023192)
Supplement: Author's manuscript [file bmjopen-2018-023192.draft_revisions.pdf]

BMJ Open

BMJ Open is committed to open peer review. As part of this commitment we make the peer review history of every article we publish publicly available.

When an article is published we post the peer reviewers' comments and the authors' responses online. We also post the versions of the paper that were used during peer review. These are the versions that the peer review comments apply to.

The versions of the paper that follow are the versions that were submitted during the peer review process. They are not the versions of record or the final published versions. They should not be cited or distributed as the published version of this manuscript.

BMJ Open is an open access journal and the full, final, typeset and author-corrected version of record of the manuscript is available on our site with no access controls, subscription charges or pay-per-view fees (http://bmjopen.bmj.com).

If you have any questions on BMJ Open's open peer review process please email editorial.bmjopen@bmj.com

BMJ Open

There's no such thing as bad publicity? Portrayals of primary care out-of-hours services in print media: A quantitative content analysis

Journal:	*BMJ Open*
Manuscript ID	bmjopen-2018-023192
Article Type:	Research
Date Submitted by the Author:	25-Mar-2018
Complete List of Authors:	Foster, Hamish; General Practice and Primary Care; University of Glasgow<br>MacDonald, Sara; University of Glasgow,<br>Patterson, Chris; University of Glasgow, MRC/CSO Social & Public Health Sciences Unit<br>O'Donnell, Catherine; University of Glasgow, General Practice & Primary Care
Keywords:	PRIMARY CARE, out-of-hours, Organisation of health services < HEALTH SERVICES ADMINISTRATION & MANAGEMENT, media reporting

SCHOLARONE™
Manuscripts

For peer review only

There's no such thing as bad publicity? Portrayals of primary care out-of-hours services in print media: A quantitative content analysis

Hamish Foster[1], Sara Macdonald[1], Chris Patterson[2], Catherine A O'Donnell[1*]

1. **General Practice & Primary Care, Institute of Health & Wellbeing, University of Glasgow.**
2. **MRC/CSO Social and Public Health Sciences Unit, Institute of Health & Wellbeing, University of Glasgow.**

Dr Hamish Foster, Clinical Academic Fellow; Hamish.Foster@glasgow.ac.uk

Dr Sara Macdonald, Senior Lecturer in Primary Care; Sara.Macdonald@glasgow.ac.uk

Mr Chris Patterson, Research Assistant; Chris.Paterson@glasgow.ac.uk

*Professor Catherine O'Donnell, Professor of Primary Care R&D; Kate.O'Donnell@glasgow.ac.uk

***Corresponding Author**

General Practice & Primary Care, Institute of Health & Wellbeing, University of Glasgow, 1 Horselethill Road, Glasgow G12 9LX. Tel: 0141 330 8329.

For peer review only

For peer review only - http://bmjopen.bmj.com/site/about/guidelines.xhtml

Abstract

Objective

To explore how out-of-hours primary healthcare services (OOHS) are portrayed in UK national newspapers, with a particular focus on the content and tone of reporting and the use of personal narratives to frame stories.

Design

Quantitative content analysis of all articles published in 2005, 2010 and 2015.

Data Sources

Nexis database used to search 10 UK national newspapers covering quality, middle-market and tabloid publications.

Inclusion/Exclusion Criteria

All articles containing the terms "out-of-hours" [≥3 mentions per article] or ("NHS 24" OR "NHS 111" OR "NHS Direct") AND "out-of-hours" [≥1 mention per article] were included. Letters, duplicate news items, opinion pieces and articles without a substantial portion of the story (>50%) concerning OOHS were excluded.

Results

332 newspaper articles were identified: 113 in 2005 (34.1%), 140 in 2010 (42.2%) and 79 in 2015 (23.8%). Of these, 195 (58.7%) were in quality newspapers; 99 (29.8%) in middle-market; 38 (11.3%) in tabloids. The most commonly reported themes were OOHS organisation, personal narratives and telephone triage. There was a predominance of stories about personal tragedy, including unsafe doctors and missed or delayed identification of rare conditions. The majority of articles (252, 75.9%) were negative in tone. This was observed for all included newspapers and by publication genre, with middle-market newspapers having the highest percentage of negative articles (Pearson

For peer review only

Chi-Square = 35.72, p<0.001). Articles presented little supporting contextual information, such as call rates per annum, or advice on how to access OOHS.

Conclusion

In this first reported analysis of national newspaper coverage of OOHS, not only is the media representation extremely negative, but it relies heavily on 'negative exemplars' of individual patient cases with little or no contextualisation. We present recommendations for the future reporting of OOHS, which could apply to the reporting of healthcare services more generally.

For peer review only

For peer review only - http://bmjopen.bmj.com/site/about/guidelines.xhtml

Strengths and Limitations

- Covers a comprehensive range of UK print media, including a representative spread of main publication genres, political leaning and readership demographics.
- Timespan covered by the analysis reflects a time of substantial change to the organisation of OOHS in the UK.
- Only newspaper articles were included.
- Other popular news sources, for example online news and social media, were excluded from this study.

For peer review only

For peer review only - http://bmjopen.bmj.com/site/about/guidelines.xhtml

Introduction

Primary care out-of-hours services (OOHS), defined as care provided when family doctors' surgeries are closed (weekdays from 1800hr to 0800hr, weekends and public holidays), have seen extensive policy and structural change in many health care systems.[1-4] In many European countries, this is characterised by a switch from small rota-based systems of primary care physicians (GPs) to regional co-operatives charged with OOHS responsibility.[5-7] In the UK, significant contractual change in 2004 offered GPs the option of transferring responsibility for OOHS to regional health organisations, leading to new models of care including out-of-hours centres, walk-in centres, and minor injuries units, as well as the implementation of national or centralised telephone triage and advice services.[3 8 9] Difficulties in recruiting family doctors to work in the out-of-hours period are one driver of these policy and structural changes.[4 7] A national audit of OOHS in England in 2014 found 60% of providers had gaps in their GP rotas.[10] In Scotland, older GPs typically contribute a disproportionate number of OOHS duty sessions, causing concern for future staffing of services.[11]

A second driver is increasing patient demand, due in part to ageing populations and associated multimorbidity,[12 13] and a concern about the supposed 'inappropriate' use of OOHS.[7] For example, a study of all out-of-hours calls to one region of Denmark between 2010-2011 found 24% of all out-of-hours calls were re-triaged as 'medically inappropriate' and could have been redirected to in-hours services.[14] Difficulties accessing daytime primary care have also been reported as leading to increased use of OOHS.[15 16] Patients have also reported uncertainty about the urgency of presenting complaints, limited knowledge about when and how to access OOHS and confusion about which services to access, all of which may drive them towards using more visible and accessible emergency departments, rather than primary care OOHS.[17 18] Thus, patients need to be able to

For peer review only - http://bmjopen.bmj.com/site/about/guidelines.xhtml

access information that helps them make decisions about where, and how, to access health care, especially for more urgent issues.

Print media remains a common source of public knowledge and a potentially powerful influence on peoples' perceptions. Newspapers are able to set public agendas, determining *what* issues are of public importance.[19] Newspapers also 'frame' issues, influencing *how* topics are viewed.[20] Negative newspaper coverage of an issue is not only associated with negative perceptions,[21] but with negative health behaviour outcomes, such as lower vaccine uptake.[22] Equally concerning are the numerous empirical examples of the misrepresentation of health issues by print media. For example, newspaper articles have been found to be overly positive in the reporting of new surgical interventions, to under-report epidemiological data in relation to the HPV vaccine,[23 24] and to report stories about rare diseases more frequently than common conditions.[25] Media analyses in the UK have also suggested that negative newspaper portrayals of GPs, and GP pay, are linked to decreasing professional morale and GP recruitment difficulties.[26 27] However, there has been no previous examination of how national UK newspapers portray OOHS despite the aforementioned significant policy and structural changes. Improved understanding of this important influence on public, and staff, perceptions may inform OOHS service providers in their patient education campaigns and in staff recruitment drives.

This study aimed to explore how UK OOHS are portrayed in national newspapers, with a particular focus on the content and tone of reporting and the use of personal narratives or individual case studies to frame stories.

For peer review only - http://bmjopen.bmj.com/site/about/guidelines.xhtml

Methods

Selection of national newspapers

Ten UK national newspapers were purposively selected to represent the breadth of UK national print media, in terms of newspaper genre (quality, middle-market or tabloid), current political alignment and readership demographics.[28-30] Newspapers included major UK and Scottish titles, and their Sunday equivalents, and comprised: *Telegraph*, *Times* and *Guardian* (UK quality publications); the *Herald* and the *Scotsman* (Scottish quality publications); the *Daily Mail* and the *Daily Express* (UK middle-market publications); the *Daily Mirror* and the *Daily Star* (UK tabloids) and the *Daily Record* (Scottish tabloid). Full details are presented in Appendix 1. Scottish titles were considered separately as, in the UK, health is a devolved matter and under the jurisdiction of the Scottish Government, with well-recognised variation in both health strategy and the organisation of service delivery.[31 32]

Searches

Searches were conducted using the Nexis database; the chosen timeframe was 1st January 2004 until 31st October 2015, which incorporated the implementation of the new General Medical Services (GMS) contract for general practitioners in the UK, with the changes to OOHS provision as previously described. All articles containing the terms "out-of-hours" [≥3 mentions per article] or ("NHS24" OR "NHS111" OR "NHSDirect" (the names of UK NHS telephone triage and advice services)) AND "out-of-hours" [≥1 mention per article] were included. Letters, duplicate news items, opinion pieces without editorial content and articles without a substantial portion of the story (>50%) concerning OOHS were excluded. The searches returned 1625 articles in total; we purposively selected all articles published in the years 2005, 2010 and 2015 for detailed content analysis, in order to provide a snapshot of reporting over this timeframe.

For peer review only - http://bmjopen.bmj.com/site/about/guidelines.xhtml

Coding and analysis

A quantitative content analysis was conducted using a coding pro-forma developed iteratively by all the authors.[30 33 34] The pro-forma recorded how often, and in which newspapers, stories about OOHS were reported; the type of story (news item, feature article, or editorial); and the main theme of the article. Main themes were identified by the study team as those relevant to the research aims, including: OOHS organisation; demand/volume of work; GP contract; and personal narrative or case study. When a personal narrative was reported, demographic details about the patient, the clinical problem presented to the OOHS and the outcome were recorded. Articles could be coded as having more than one theme, for example service organisation and a personal narrative.

HF, SM and CO'D read and coded a total of 100 articles using the pro-forma; all authors discussed the findings and refined the pro-forma, adding and refining themes as identified (e.g. whether articles used supporting statistics or gave advice to readers about accessing OOHS). Following that, the same authors each selected one identified year (2005, 2010 or 2015) and coded the remaining articles from that year.

Data were entered into STATA for statistical analysis. Textual data on the themes contained in the articles were coded numerically (1 = yes, theme was reported; 2 = no, theme was not reported); thus, the resulting analysis was quantitative.[30] Much of the coding recorded the presence or absence of thematic content, e.g. did the article report on the GP contract or not. However, some coding required interpretation of the meaning underlying surface content, described as latent coding,[30 33] such as when recording the tone of an article. Each article's overall tone was coded as being positive, negative or neutral; this was assessed by the researcher coding that paper and discussed with the rest of the research team. Kruskal-Wallis tests were used to test the statistical

For peer review only - http://bmjopen.bmj.com/site/about/guidelines.xhtml

significance of relationships between article tone and publication; article tone and publication genre; and variation in median article word count and publication genre. A Chi-square test was used to test differences in tone between publication genres, while a Wilcoxon signed-rank test was used to determine whether publications' media tone deviated significantly from a neutral tone. The threshold for statistical significance was set at p<0.01.

Patient Involvement

The stimulus for this study came from work conducted for the Scottish Government's Out-of-Hours Review Group, which included a range of policy, professional and patient stakeholder groups. However, patients were not explicitly involved in the design or interpretation of the work reported here.

Results

There were 332 articles from the sampled years: 113 (34.1%) published in 2005; 140 (42.2%) in 2010; and 79 (23.8%) in 2015. Overall 182 (54.8%) articles were news reports; 44 (13.3%) were features; and 99 (29.8%) were editorials. Of the 332 articles, 195 (58.7%) were in the quality press; 99 (29.8%) in middle-market newspapers; and 38 (11.5%) in the tabloids. While there was variation across publications and by year, the *Daily Mail/Mail on Sunday* published the most articles (n=76 (22.9% of total)) whereas the *Daily Star/Daily Star Sunday* published just one article (Figure 1). The *Guardian/Observer* and *Daily Telegraph/Sunday Telegraph* both published significantly more articles on OOHS in 2010 than in either 2005 or 2015.

The overall median word count was 508.0 words (25th to 75th percentiles: 323.0 to 687.5). Median word count was highest in the middle-market newspapers and lowest in the tabloids (Quality:

For peer review only - http://bmjopen.bmj.com/site/about/guidelines.xhtml

median 491.0 (25th to 75th percentiles: 329.0 to 654.0); Middle-market: median 612.0 (25th to 75th percentiles: 445.0 to 804.0; Tabloid: median 336.5 (25th to 75th percentiles: 199.3 to 504.0) (Kruskall-Wallis $p<0.001$).

Thematic content

Thematic content was coded 621 times across the 332 articles, thus most articles reported more than one theme. The most frequently reported themes were service organisation, personal narratives, and telephone triage (Table 1), but there were variations by year and publication genre. In 2005, telephone triage was a recurrent theme; 53 of 60 references to telephone triage in 2005 related to a widely publicised report on NHS 24, the then relatively new Scottish telephone triage service, following a fatal accident enquiry. The theme of telephone triage in 2005 also linked to the service organisation theme, because articles reporting telephone triage often discussed wider OOHS organisational issues. Articles reporting personal narratives were second most frequent in 2010 (n=83), prompting closer examination of these articles (see below). Broadly similar frequencies of topics were seen across the three publication genres, but tabloid newspapers published fewer articles concerned with service organisation.

Articles coded as ‘other’ encompassed a range of topics, including service response times; patient satisfaction; GP OOH responsibility; confusion around services; and quality of care. In each case, there were only 1 to 5 articles concerned with each topic. The exception was the theme of ‘unsafe non-UK doctors’, which was a recurrent theme in 2010, comprising 58 of the 83 articles coded as ‘other’. Inspection of these 58 articles found that 53 (91.4%) of these occurrences were in newspaper articles also coded to the theme of ‘personal narrative’. This is presented in more detail in the section entitled Reporting of personal narratives.

For peer review only - http://bmjopen.bmj.com/site/about/guidelines.xhtml

Only 12 (3.6%) of OOHS news articles mentioned any explicit self-management advice for patients or offered information on how best to use OOHS, and only 1 article contained advice on accessing OOHS. Conversely, 26 articles (7.8%) described confusion around OOHS. Numerical data (e.g. focused on reduced numbers of staff on duty, large number of calls or home visits delayed, high annual costs) were frequently cited in articles but denominator or contextual data was rarely given alongside the headline statistic, for example the call rate per annum or number of cases of meningitis seen by an OOHS in one year.

Article Tone

Overall, 252 of the 332 articles (75.9%) were negative with only 11 (3.3%) positive, and 69 (20.1%) neutral (Table 2). Articles published in the middle-market newspapers were more likely to be negative in tone (86/99; 86.9%) compared with the quality newspapers (136/195; 69.7%) or the tabloids (30/38; 79.0%) (Pearson Chi-Square = 35.72, p<0.001). Each individual publication leaned significantly toward a negative tone (Wilcoxon signed-rank p<0.01). While no publication was significantly more positive/negative/neutral than any other (Kruskall-Wallis p=0.025) some – notably the Scottish quality newspapers – tended towards a more neutral tone of reporting (Table 2). Tone also varied by year, with 69.9%, 90.7% and 58.2% of articles negative in tone for years 2005, 2010, and 2015 respectively.

Reporting of personal narratives

Nearly half of the articles (153; 46.1%) mentioned at least one personal narrative, with the majority portraying such stories as tragedies (132/153; 86.3%). Most featured the personal narrative as the main theme (108/153; 70.6%); however, others commented on a personal narrative in passing, for example at the end of an article. Where personal narrative was a main theme, the majority described narratives that involved rare diagnoses or problems (104/108; 96.3%). Examples included

For peer review only - http://bmjopen.bmj.com/site/about/guidelines.xhtml

fatal iatrogenic overdose, fatal sepsis or fatal meningitis in younger patients. Individual cases were also repeated in multiple stories. This was particularly apparent in one story, reported in 2010, of an overseas doctor working in an OOHS who accidently administered a fatal overdose of diamorphine to a patient during the doctor's first OOHS shift in the UK. Of the 140 articles on OOHS published in 2010, 89 (63.6%) referenced this case. Of these 89 articles, 58 (65.2%) were coded to the theme of 'unsafe non-UK doctors' – meaning that a main theme of these articles was this story. The remaining 31 articles used this story indirectly to illustrate a point in an unrelated article.

Discussion

This is the first study that describes UK newspaper portrayal of OOHS. Our main finding is that articles on OOHS were significantly more likely to have a negative tone than a positive or neutral tone; middle-market newspapers were significantly more likely to have a negative tone compared with the quality or tabloid newspapers. These findings highlight an over-representation of negative stories around OOHS, as illustrated by the word cloud of the most frequently occurring 'negative' headline words (Figure 2). This negativity is unrelated to newspaper political slant or readership demographics. This representation of OOHS contrasts with patient views of care, with the 2017 GP patient survey in England finding that 66.2% of respondents who had recently accessed OOHS rated their overall experience as 'fairly or very good'.[35] There has, however, been a slight drop in satisfaction since the question was first asked in the 2012 survey, where 70.9% of respondents replied positively.[36] While this slight decrease cannot be attributed to media coverage, it is part of a pattern. Two previous studies of GP portrayal and GP pay identified a change in GP newspaper portrayal over time related to contractual change, with portrayals of both GPs and their salaries becoming more negative after the introduction of the 2004 GMS contract.[26 27] However, our study

For peer review only

For peer review only - http://bmjopen.bmj.com/site/about/guidelines.xhtml

found much higher levels of negativity, in terms of reporting, than either of the two previous studies.[26] This may have implications for patients' on-going trust of both OOHS, and GPs in general.

Our analysis also identified the important role that a single case can play in framing media representation of OOHS. It is important to acknowledge that some cases will indeed be worth reporting and a missed diagnosis resulting in a death is always a tragic incident, however such incidents a rare occurrences. Our argument, however, is that the constant focus and repeated reporting of particular cases provides a skewed picture of OOHS. For example, the reporting in 2010 of the Coroner's ruling on the unlawful killing of a patient by a doctor working in an OOHS dominated the reporting that year. More OOHS articles were published in 2010 than the other two years, and a large proportion of them reported on this case either directly or indirectly. In addition, a higher proportion of articles published in 2010 were negative, compared with the other two years sampled. This case was often included in unrelated articles as an exemplar of an OOHS tragedy. This use of 'negative exemplars' was also seen in relation to personal narratives about the presentation of rare clinical problems, such as septicaemia or meningitis, with such cases often used in passing at the end of unrelated OOHS articles. This is in line with previous media analysis that showed leading US magazines and newspapers over-represented infrequent causes of death while under-representing common causes.[25] Public perception of the safety of OOHS could therefore be skewed by an over-representation of rare and tragic cases. This is coupled with a lack of reliable statistical reporting and advice on when, and how, to access OOHS when required. Doctors may also be reluctant to work in OOHS, where they may be concerned about managing risk in undifferentiated presentations of acute illness and the possible consequences if they are judged to 'make a mistake'. While the media have an important role in holding public bodies and services to account, the media also has a responsibility to portray fair representations of public services and to provide impartial

For peer review only - http://bmjopen.bmj.com/site/about/guidelines.xhtml

information. Misrepresentations may affect people's interaction with, and outcomes from, OOHS either by increasing demand through unnecessary concern over rare illnesses or, alternatively, through eroding public trust and therefore delaying use of the service.

Despite reporting on confusion around OOHS services, newspapers offered little practical guidance on accessing care. Thus, to counteract skewed representations of OOHS and their staff, to reduce inappropriate OOHS use and to improve public understanding of health services we recommend the development of guidelines for media reporting on health services. While the starting point here is OOHS, these could be adapted to other healthcare settings.

Developing reporting guidelines for OOHS

National and international guidelines already exist for the media reporting of suicides,[37 38] with the threat of failure to adhere to such guidelines in the UK resulting in referral to the UK Press Complaints Commission. Best practice guidelines also exist for UK media reporting of scientific studies.[39] However, these guidelines focus on research findings and omit recommendations specific to health services that are of public health importance. Box 1 contains suggested recommendations on OOHS reporting that could be integrated into current reporting guidelines.

For peer review only - http://bmjopen.bmj.com/site/about/guidelines.xhtml

1. Media reporting of OOHS must stop the practice of adding a 'negative exemplar' at the end of a story unrelated to that personal narrative.
2. Media sources are encouraged to consider adopting the practice of 'positive exemplars', namely personal narratives where someone received timely and appropriate care, perhaps for a rare or unusual clinical problem.
3. Where personal narratives are reported to depict poor care and specific diseases or illnesses are mentioned, provide further disease-related information or direct readers to further information from providers of impartial health advice. For example, on reporting following a case of fatal sepsis in a young person direct the reader to the website of a prominent sepsis charity/organisation.
4. When reporting on service access problems direct readers to further information on how and when to navigate services. For example, if reporting on a case of delay in care direct the reader to NHS Choices or provide the local telephone triage service number.
5. When reporting health service related statistics provide contextual data. For example, if reporting on how few doctors are covering an OOHS shift, provide information or provide links to information on how many staff normally cover that shift. Or if reporting on costs of a service direct readers to more information on costs of other public services.
6. When reporting on use of an OOHS, provide contextual information such as the number of calls or visits the service received per annum.
7. Where possible, provide links to local information or campaigns about alternative sources of health care support e.g. local pharmacies.

Box 1. Media reporting guidelines for articles reporting on OOHS.

Strengths and limitations

This is the first media analysis of reporting on OOHS services. The analysis covered a comprehensive range of UK print media, including a representative spread of main publication genres, political leaning and readership demographics. The timespan covered by the analysis reflects a time of substantial change to the organisation of OOHS in the UK. The study only included newspaper articles, and there are likely to be other popular news sources excluded from this study that also exert influence on public perceptions, for example online news and social media. However, while print newspaper circulation is declining, online news is largely dominated by the online

For peer review only - http://bmjopen.bmj.com/site/about/guidelines.xhtml

counterparts of traditional newspapers, such that analysing online news content may yield similar results.

Conclusion

Newspaper reports on OOHS were generally negative irrespective of newspaper type. OOHS articles frequently described personal narratives depicting rare and tragic patient stories, even when the main story was unrelated to the personal narrative. In 2010, reporting frequently focused on 'unsafe non-UK doctors' due to a single personal narrative. Our findings provide clear examples of unhelpful media misrepresentation that may negatively affect the public's perceptions of, and interaction with, OOHS. Developing guidelines to encourage responsible reporting on health services may have a role in reducing the risk of skewed public perceptions. Further research that examines public perceptions of OOHS in light of these newspaper representations would develop understanding of the media's role in shaping public opinion of health services.

For peer review only - http://bmjopen.bmj.com/site/about/guidelines.xhtml

Contributors.

HF, SM and COD conceived the idea and designed the study; HF and CP designed the search strategies, with input from SM and COD. All authors contributed to the design of the data extraction pro-forma and data extraction. HF and CP analysed the data; all authors contributed to data interpretation. HF wrote the first draft; COD led the re-drafting; all authors contributed to the final version and agreed to its submission. COD is the guarantor.

Funding.

This study was unfunded. HF is supported by an NHS Education for Scotland Academic Fellowship. CP's time was funded by the Informing Healthy Public Policy programme (Funded by the Medical Research Council MC_UU_12017–15 and the Chief Scientist Office SPHSU15) of the MRC/CSO Social and Public Health Sciences Unit, University of Glasgow.

Competing interests.

All authors have completed the ICMJE uniform disclosure form at http://www.icmje.org/coi_disclosure.pdf and declare: no support from any organisation for the submitted work; no financial relationships with any organisations that might have an interest in the submitted work in the previous three years, no other relationships or activities that could appear to have influenced the submitted work.

Ethical approval.

Ethical approval was not required for this study.

Copyright/License for publication.

For peer review only - http://bmjopen.bmj.com/site/about/guidelines.xhtml

The Corresponding Author has the right to grant on behalf of all authors and does grant on behalf of all authors, a worldwide licence to the Publishers and its licensees in perpetuity, in all forms, formats and media (whether known now or created in the future), to i) publish, reproduce, distribute, display and store the Contribution, ii) translate the Contribution into other languages, create adaptations, reprints, include within collections and create summaries, extracts and/or, abstracts of the Contribution, iii) create any other derivative work(s) based on the Contribution, iv) to exploit all subsidiary rights in the Contribution, v) the inclusion of electronic links from the Contribution to third party material where-ever it may be located; and, vi) licence any third party to do any or all of the above.

For peer review only

References

1. Hurwitz B. The new out of hours agreement for general practitioners. *British Medical Journal* 1995;311:824-25.

2. Hansen BL, Munck A. Out-of-hours service in Denmark: The effect of a structural change. *British Journal of General Practice* 1998;48(433):1497-99.

3. Tan S, Mays N. Impact of initiatives to improve access to, and choice of, primary and urgent care in the England: A systematic review. *Health Policy* 2014;118(3):304-15.

4. Smits M, Rutten M, Keizer E, et al. The development and performance of after-hours primary care in the Netherlands: A narrative review. *Annals of Internal Medicine* 2017;166:737-42. doi: 10.7326/M16-2776

5. Christensen MB, Olesen F. Out of hours service in Denmark: evaluation five years after reform. *BMJ* 1998;316:1502-05.

6. van Uden CJT, Giesen PHJ, Metsemakers JFM, et al. Development of out-of-hours primary care by general practitioners (GPs) in The Netherlands: from small-call rotations to large-scale GP cooperatives. *Family Medicine* 2006;38(8):565-69.

7. Berchet C, Nader C. The organisation of out-of-hours primary care in OECD countries: OECD Publishing, 2016.

8. Munro JF, Nicholl JP, O'Cathain A, et al. Impact of NHS Direct on demand for immediate care: observational study. *BMJ* 2000;321:150-53.

9. Turner J, O'Cathain A, Knowles E, et al. Impact of the urgent care telephone service NHS 111 pilot sites: a controlled before and after study. *BMJ Open* 2013;3:e003451.

10. National Audit Office. Out-of-hours GP services in England, 2014.

11. The Scottish Government. Pulling together: transforming urgent care for the people of Scotland. The Report of the Independent Review of Primary Care Out of Hours Services: National Review of Priary Care Out of Hours Services. A Scottish Government commisioned report, 2015.

For peer review only

For peer review only - http://bmjopen.bmj.com/site/about/guidelines.xhtml

12. Barnett K, Mercer SW, Norbury M, et al. Epidemiology of multimorbidity and implications for health care, research, and medical education: a cross-sectional study. *Lancet* 2012;380(9836):37-43. doi: 10.1016/S0140-6736(12)60240-2

13. Hobbs FDR, Bankhead C, Mukhtar T, et al. Clinical workload in UK primary care: a retrospective analysis of 100 million consultations in England, 2007-14. *Lancet* 2016;387(10035):2323-30. doi: 10.1016/S0140-6736(16)00620-6

14. Noroxe KB, Huibers L, Moth G, et al. Medical appropriateness of adult calls to Danish out-of-hours primary care: a questionnaire-based survey. *BMC Fam Pract* 2017;18(1):34. doi: 10.1186/s12875-017-0617-1

15. Zhou Y, Abel G, Warren F, et al. Do difficulties in accessing in-hours primary care predict higher use of out-of-hours GP services? Evidence from an English National Patient Survey. *Emergency Medicine Journal* 2015;32(5):373-78.

16. Smits M, Peters Y, Broers S, et al. Association between general practice characteristics and use of out-of-hours GP cooperatives. *BMC Family Practice* 2015;16(1):52.

17. MacKichan F, Brangan E, Wye L, et al. Why do patients seek primary medical care in emergency departments? An ethnographic exploration of access to general practice. *BMJ Open* 2017;7 doi: 10.1136/bmjopen-2016-013816

18. Weber EJ, Hirst E, Marsh M. The patient's dilemma: attending the emergency department with a minor illness. *BMJ* 2017;357:j1941. doi: 10.1136/bmj.j1941

19. Shaw DL, Martin SE. The Function of mass-media agenda setting. *Journalism Quart* 1992;69(4):902-20.

20. Scheufele DA. Framing as a theory of media effects. *J Commun* 1999;49(1):103-22. doi: DOI 10.1111/j.1460-2466.1999.tb02784.x

21. Nagelhout GE, van den Putte B, de Vries H, et al. The influence of newspaper coverage and a media campaign on smokers' support for smoke-free bars and restaurants and on secondhand smoke harm awareness: findings from the International Tobacco Control (ITC) Netherlands Survey. *Tobacco Control* 2012;21(1):24-9. doi: 10.1136/tc.2010.040477

For peer review only

For peer review only - http://bmjopen.bmj.com/site/about/guidelines.xhtml

22. Mason BW, Donnelly PD. Impact of a local newspaper campaign on the uptake of the measles mumps and rubella vaccine. *Journal of Epidemiology and Community Health* 2000;54(6):473-74. doi: DOI 10.1136/jech.54.6.473

23. Malviya A, Stafford GH, Villar RJF, et al. Have the media influenced the use of hip resurfacing arthroplasty? A review of UK print media. *Ann Roy Coll Surg* 2012;94(6):432-37. doi: 10.1308/003588412x13171221592014

24. Bodemer N, Müller SM, Okan Y, et al. Do the media provide transparent health information? A cross-cultural comparison of public information about the HPV vaccine. *Vaccine* 2012;30(25):3747-56. doi: https://doi.org/10.1016/j.vaccine.2012.03.005

25. Frost K, Frank E, Maibach E. Relative risk in the news media: A quantification of misrepresentation. *Am J Public Health* 1997;87(5):842-45. doi: Doi 10.2105/Ajph.87.5.842

26. Balkham A, Alderson S. 'The biggest car crash in NHS history': the media portrayal of GP pay before and after the introduction of the Health and Social Care Bill 2011. *Primary Health Care Research & Development* 2017;18(1):84-91. doi: 10.1017/S1463423616000359

27. Tanner F, Foy R, Harrison W. 'Wads up, Doc' - trends in British newspapers' reporting of general practitioners' pay. *Primary Health Care Research & Development* 2010;11(4):405-09.

28. Hilton S, Patterson C, Teyhan A. Escalating coverage of obesity in UK newspapers: The evolution and framing of the "obesity epidemic" from 1996 to 2010. *Obesity* 2012;20(8):1688-95. doi: 10.1038/oby.2012.27

29. Rooke C, Amos A. News media representations of electronic cigarettes: an analysis of newspaper coverage in the UK and Scotland. *Tobacco Control* 2014;23(6):507-12. doi: 10.1136/tobaccocontrol-2013-051043

30. Patterson C, Katikireddi SV, Wood K, et al. Representations of minimum unit pricing for alcohol in UK newspapers: a case study of a public health policy debate. *Journal of Public Health* 2015;37(1):40-49. doi: 10.1093/pubmed/fdu078

For peer review only - http://bmjopen.bmj.com/site/about/guidelines.xhtml

31. Bevan G, Karanikolos M, Exley J, et al. The four health systems of the United Kingdon: how do they compare? London, 2014.

32. Greer SL. Devolution and health in the UK: policy and its lessons since 1998. *British Medical Bulletin* 2016;118(1):16-24. doi: 10.1093/bmb/ldw013

33. Downe-Wamboldt B. Content analysis: method, applications, and issues. *Health Care for Women International* 1992;13(3):313-21. doi: 10.1080/07399339209516006

34. Manganello J, Blake N. A study of quantitative content analysis of health messages in U.S. media from 1985 to 2005. *Health Communication* 2010;25(5):387-96. doi: 10.1080/10410236.2010.483333

35. Ipsos MORI, NHS England. GP patient survey 2017. National results and trends: NHS England, 2017.

36. Ipsos MORI, England N. The GP patient survey 2011/2012. Summary report, 2012.

37. The Samaritans. Media guidelines for reporting suicide: The Samaritans, 2017.

38. World Health Organization, International Association for Suicide Prevention. Preventing suicide. A resource for media professionals, 2008.

39. Science Media Centre. 10 best practice guidelines for reporting science and health stories: Science Media Centre, 2012.

For peer review only

For peer review only - http://bmjopen.bmj.com/site/about/guidelines.xhtml

Table 1. Reporting of OOHS themes by year and by publication genre (Number of times theme coded (%))*

Theme	Total number of times theme coded (n = 621)	Publication year (n, %)			Publication genre (n, %)		
		2005 (n = 174)	2010 (n = 332)	2015 (n = 115)	Quality (n = 365)	Middle-market (n = 192)	Tabloid (n = 64)
Service organisation	139	40 (23.0)	75 (22.6)	24 (20.9)	92 (29.5)	40 (25.8)	7 (10.9)
Case study/Personal narrative	108	13 (7.5)	83 (25.0)	12 (10.4)	62 (19.9)	33 (21.3)	13 (20.3)
Telephone triage	95	60 (34.3)	11 (3.3)	24 (20.9)	59 (18.9)	21 (13.5)	15 (23.4)
GP contract	52	18 (10.3)	30 (9.0)	4 (3.5)	25 (8.0)	25 (16.1)	2 (3.1)
Demand/Volume of work	44	16 (9.2)	10 (3.0)	18 (15.7)	28 (9.0)	12 (7.7)	4 (6.2)
GP pay	33	12 (6.9)	17 (5.1)	4 (3.5)	15 (4.8)	13 (8.5)	5 (7.8)
Rurality	20	3 (1.7)	15 (4.5)	2 (1.7)	17 (5.4)	0 (0.0)	3 (4.7)
Seven day services	12	2 (1.2)	1 (0.3)	9 (7.8)	4 (1.3)	7 (4.5)	1 (1.6)
Public responsibility/Appropriateness	10	1 (0.6)	3 (0.9)	6 (5.2)	4 (1.3)	2 (1.3)	4 (6.2)
Cost of services	8	2 (1.2)	4 (1.3)	2 (1.7)	6 (1.9)	2 (1.3)	0 (0.0)
Other	100	7 (4.0)	83 (25.0)	10 (8.7)	53 (17.0)	37 (23.9)	10 (15.7)

*Articles coded to more than 1 theme

For peer review only - http://bmjopen.bmj.com/site/about/guidelines.xhtml

Table 2. Tone by newspaper publication (Number of articles, (% within newspaper publication))

Publication	Positive tone (n=11)	Negative tone (n=252)	Neutral tone (n=69)
**Quality**			
Herald/Sunday Herald (n=51)	1 (2.0)	30 (58.8)	20 (39.2)
Times/Sunday Times (n=40)	0 (0.0)	32 (80.0)	8 (20.0)
Daily Telegraph/Sunday Telegraph (n=35)	1 (2.9)	26 (74.3)	8 (22.8)
Guardian/Observer (n=38)	2 (5.3)	29 (76.3)	7 (18.4)
Scotsman/Scotland on Sunday (n=31)	1 (3.2)	19 (61.3)	11 (35.5)
**Middle-Market**			
Daily Mail/Mail on Sunday (n=76)	0 (0.0)	67 (88.2)	9 (11.8)
Express/Sunday Express (n=23)	0 (0.0)	19 (82.6)	4 (17.4)
**Tabloid**			
Daily Record/Sunday Mail (n=25)	5 (20.0)	19 (76.0)	1 (4.0)
Daily Mirror/Sunday Mirror (n=12)	1 (8.3)	10 (83.3)	1 (8.3)
Daily Star/Daily Star Sunday (n=1)	0 (0.0)	1 (100.0)	0 (0.0)

For peer review only

For peer review only - http://bmjopen.bmj.com/site/about/guidelines.xhtml

Figure 1. Article frequency by publication and year.

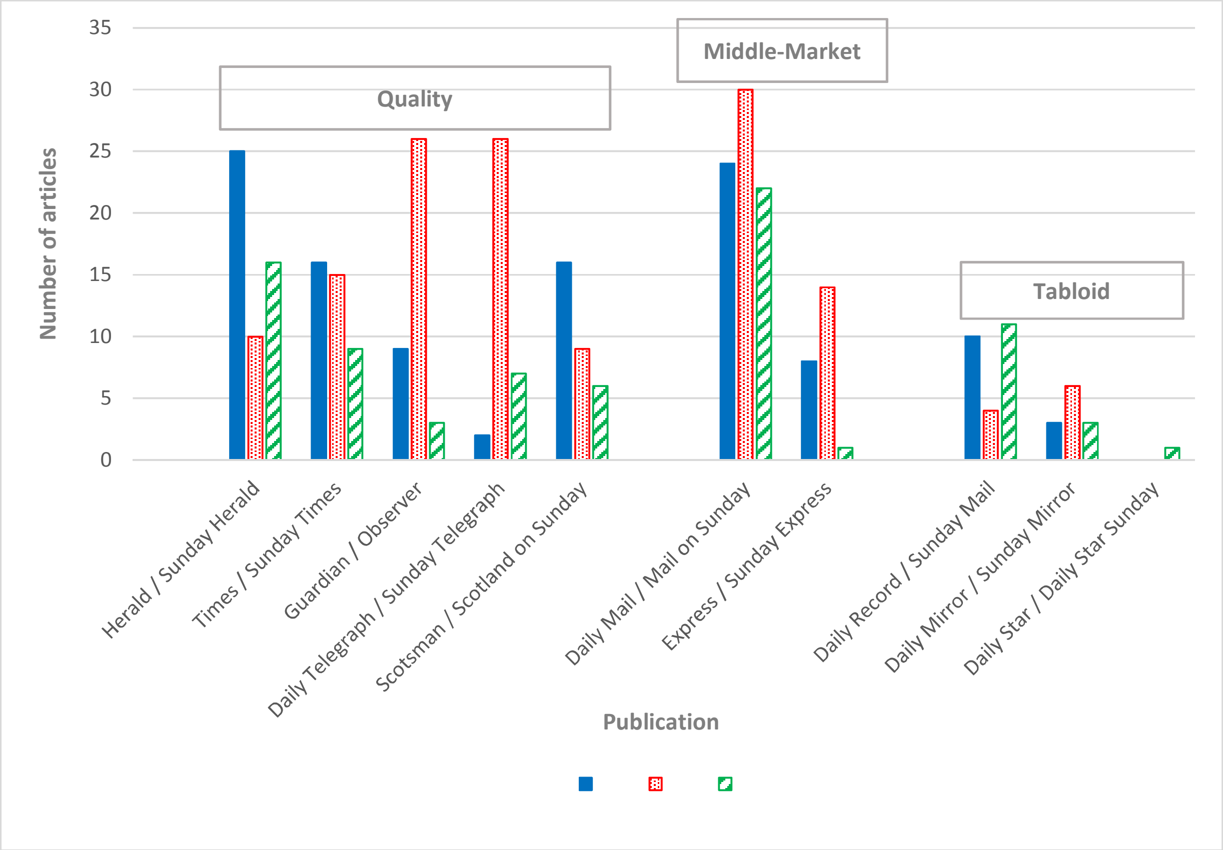

Figure 2. Word cloud of most frequently appearing negative words in headlines about OOHS services.

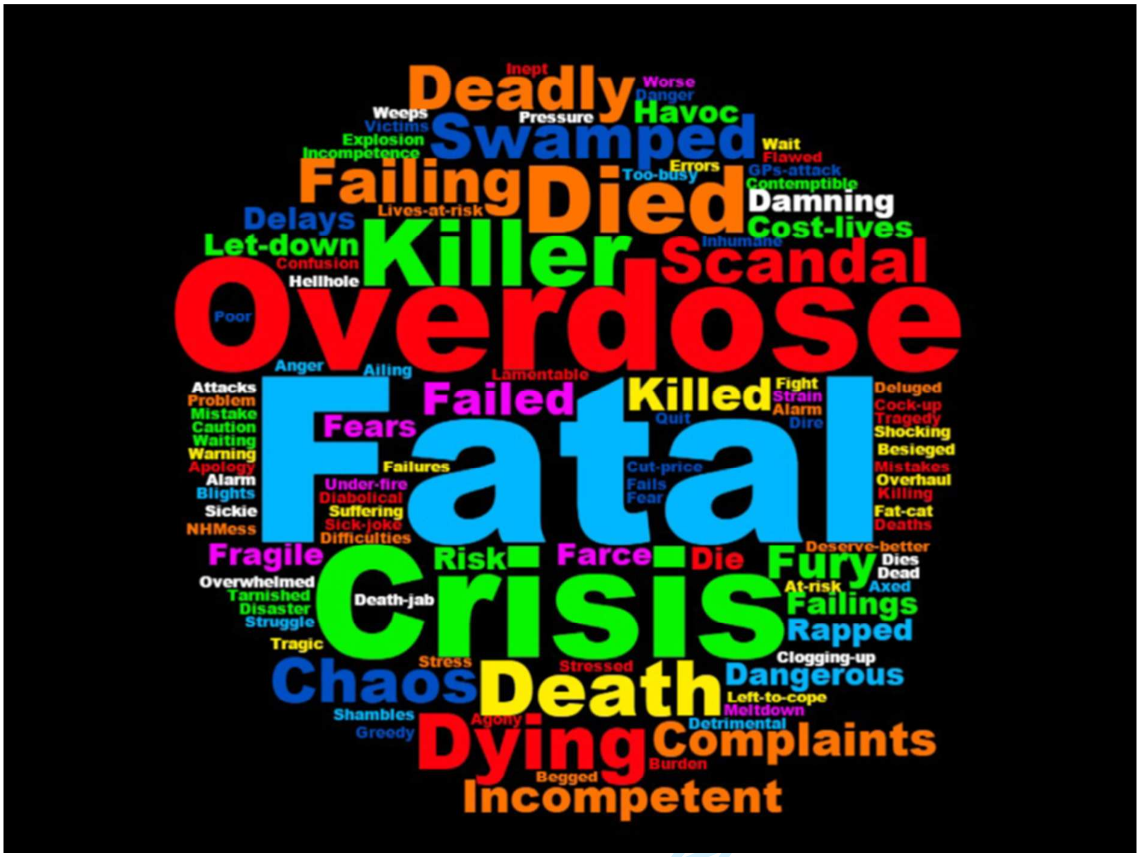

w only

Appendix 1. Overview of main UK and Scottish daily and Sunday newspapers selected by publication location, genre, circulation, readership and political ideology, October 205 – September 2016. (From: National Readership Survey, http://www.nrs.co.uk/downloads/pdf/newspapers_201611.pdf)

Newspaper	Estimated weekly readership (Number)	% Readership Female	% Readership in Social Group C2DE	% Readership Aged 35 +	Political Ideology
**UK**					
**Quality**					
Daily Telegraph	1,183,000	47.4	17.8	89.6	Right
The Sunday Telegraph	1,113,000	46.9	15.5	89.2	Right
The Times	1,047,000	44.6	15.2	83.8	Centre-right
The Sunday Times	1,834,000	47.0	15.4	83.9	Centre-right
The Guardian	865,000	48.4	20.1	73.9	Centre-left
The Observer	764,000	45.8	13.2	80.9	Centre-left
**Middle-Market**					
Daily Mail	3,354,000	53.9	38.0	87.7	Right
The Mail on Sunday	3,608,000	51.5	39.2	87.0	Right
Daily Express	842,000	48.8	43.6	91.8	Right
Sunday Express	826,000	47.5	40.4	88.0	Right
**Tabloid**					
The Sun*	4,188,000	42.7	68.3	75.5	Right
The Sun on Sunday*	3,640,000	44.6	65.3	72.4	Right
Daily Mirror	1,818,000	46.1	65.0	83.7	Left
Sunday Mirror	1,804,000	49.8	61.5	79.0	Left
Daily Star	800,000	34.5	75.1	74.8	Neutral
Daily Star Sunday	584,000	40.8	74.5	71.2	Neutral

For peer review only

**Scotland**					
**Quality**					
The Herald	109,000	33.0	26.6	87.2	Neutral
Sunday Herald	109,000	46.8	30.3	73.4	Neutral
The Scotsman	73,000	42.5	21.9	87.7	Neutral
Scotland on Sunday	72,000	51.4	18.1	84.7	Neutral
**Tabloid**					
Daily Record	484,000	48.3	69.4	89.1	Left
Sunday Mail	558,000	50.7	67.0	89.1	Left

*Not included in this analysis, as The Sun is not included in the Nexis database; included here for comparison.

For peer review only

Foster, Macdonald, Patterson & O'Donnell. There's no such thing as bad publicity? Portrayals of primary care out-of-hours services in print media: A quantitative content analysis

Please Note: We were unable to find a checklist suitable for a quantitative content analysis of media reporting, so have adapted the STROBE checklist accordingly.

	Item No.	STROBE items	Location in manuscript where items are reported
	1	(a) Indicate the study's design with a commonly used term in the title or the abstract (b) Provide in the abstract an informative and balanced summary of what was done and what was found	In title: There's no such thing as bad publicity? Portrayals of primary care out-of-hours services in print media: A quantitative content analysis.<br><br>Study design and description are also included in the abstract, as follows:<br><br>**Design:** Quantitative content analysis of all articles published in 2005, 2010 and 2015.<br>**Data Sources**<br>Nexis database used to search 10 UK national newspapers covering quality, middle-market and tabloid publications.<br>**Inclusion/Exclusion Criteria**<br>All articles containing the terms "out-of-hours" [≥3 mentions per article] or ("NHS 24" OR "NHS 111" OR "NHS Direct") AND "out-of-hours" [≥1 mention per article] were included. Letters, duplicate news items, opinion pieces and articles without a substantial portion of the story (>50%) concerning OOHS were excluded.<br>**Results**<br>332 newspaper articles were identified: 113 in 2005 (34.1%), 140 in 2010 (42.2%) and 79 in 2015 (23.8%). Of these, 195 (58.7%) were in quality newspapers; 99 (29.8%) in middle-market; 38 (11.3%) in tabloids. The most commonly reported themes were OOHS organisation, personal narratives and telephone triage. There was a predominance of stories about personal tragedy, including unsafe doctors and missed or delayed identification of rare conditions. The majority of articles (252, 75.9%) were negative in tone. This was observed for all included newspapers and by publication genre, with middle-market newspapers having the highest percentage of negative articles (Pearson Chi-Square = 35.72, p<0.001). Articles presented little supporting contextual information, such as call rates per annum, or advice on how to access OOHS.
Background	2	Explain the scientific background	The scientific background and rationale are given in the introduction. The study aim

rationale		and rationale for the investigation being reported	is stated on page 6:<br><br>This study aimed to explore how UK OOHS are portrayed in national newspapers, with a particular focus on the content and tone of reporting and the use of personal narratives or individual case studies to frame stories.
Objectives	3	State specific objectives, including any prespecified hypotheses	As per (2), study aim is given on page 6:<br><br>This study aimed to explore how UK OOHS are portrayed in national newspapers, with a particular focus on the content and tone of reporting and the use of personal narratives or individual case studies to frame stories.
Study Design	4	Present key elements of study design early in the paper	Study design is first described in the abstract:<br><br>**Design:** Quantitative content analysis of all articles published in 2005, 2010 and 2015.<br>**Data Sources**<br>Nexis database used to search 10 UK national newspapers covering quality, middle-market and tabloid publications.<br>**Inclusion/Exclusion Criteria**<br>All articles containing the terms "out-of-hours" [≥3 mentions per article] or ("NHS 24" OR "NHS 111" OR "NHS Direct") AND "out-of-hours" [≥1 mention per article] were included. Letters, duplicate news items, opinion pieces and articles without a substantial portion of the story (>50%) concerning OOHS were excluded.<br><br>These elements are expanded on in the Methods section (pages 7 to 9), which covers: Selection of national newspapers; Searches; Coding and analysis.
Setting	5	Describe the setting, locations, and relevant dates, including periods of recruitment, exposure, follow-up, and data collection	This study did not involve patient or participant recruitment.<br><br>Newspapers selected, searches and timeframe for the years of analysis are all described in the Methods, pages 7 to 9.
Participants	6	*(a) Cohort study* - Give the eligibility criteria, and the sources and methods of selection of participants. Describe methods of follow-up<br>*Case-control study* - Give the eligibility criteria, and the sources and methods of case ascertainment and control selection. Give the rationale for the choice of cases and controls	Not applicable.

		Cross-sectional study - Give the eligibility criteria, and the sources and methods of selection of participants<br><br>*(b) Cohort study* - For matched studies, give matching criteria and number of exposed and unexposed<br>*Case-control study* - For matched studies, give matching criteria and the number of controls per case	 <br><br><br>Not applicable.
Variables	7	Clearly define all outcomes, exposures, predictors, potential confounders, and effect modifiers. Give diagnostic criteria, if applicable.	Content analysis addressed word count, reporting themes, tone of article contents and, if applicable, details on the reporting o personal narratives.
Data sources/ measurement	8	For each variable of interest, give sources of data and details of methods of assessment (measurement).<br>Describe comparability of assessment methods if there is more than one group	In Methods, pages 8 to 9:<br><br>Textual data on the themes contained in the articles were coded numerically (1 = yes, theme was reported; 2 = no, theme was not reported); thus, the resulting analysis was quantitative.[30] Much of the coding recorded the presence or absence of thematic content, e.g. did the article report on the GP contract or not. However, some coding required interpretation of the meaning underlying surface content, described as latent coding, [30 33] such as when recording the tone of an article. Each article's overall tone was coded as being positive, negative or neutral; this was assessed by the researcher coding that paper and discussed with the rest of the research team. Kruskal-Wallis tests were used to test the statistical significance of relationships between article tone and publication; article tone and publication genre; and variation in median article word count and publication genre. A Chi-square test was used to test differences in tone between publication genres, while a Wilcoxon signed-rank test was used to determine whether publications' media tone deviated significantly from a neutral tone. The threshold for statistical significance was set at $p<0.01$.
Bias	9	Describe any efforts to address potential sources of bias	In Methods:<br><br>A quantitative content analysis was conducted using a coding pro-forma developed iteratively by all the authors.[30 33 34] The pro-forma recorded how often, and in which newspapers, stories about OOHS were reported; the type of story (news item, feature

For peer review only - http://bmjopen.bmj.com/site/about/guidelines.xhtml

			article, or editorial); and the main theme of the article. Main themes were identified by the study team as those relevant to the research aims, including: OOHS organisation; demand/volume of work; GP contract; and personal narrative or case study. When a personal narrative was reported, demographic details about the patient, the clinical problem presented to the OOHS and the outcome were recorded. Articles could be coded as having more than one theme, for example service organisation and a personal narrative.<br>HF, SM and CO'D read and coded a total of 100 articles using the pro-forma; all authors discussed the findings and refined the pro-forma, adding and refining themes as identified (e.g. whether articles used supporting statistics or gave advice to readers about accessing OOHS). Following that, the same authors each selected one identified year (2005, 2010 or 2015) and coded the remaining articles from that year.
Study size	10	Explain how the study size was arrived at	A purposive sample of three years was selected, over the time-period 2004 to 2015. We selected three years for analysis: 2005, 2010 and 2015. These years were selected to cover a period of change and reorganisation in out-of-hours care, which began following the implementation of the General Medical Services contract in 2004 in the UK.
Quantitative variables	11	Explain how quantitative variables were handled in the analyses. If applicable, describe which groupings were chosen, and why	In Methods, pages 8 to 9:<br><br>Data were entered into STATA for statistical analysis. Textual data on the themes contained in the articles were coded numerically (1 = yes, theme was reported; 2 = no, theme was not reported); thus, the resulting analysis was quantitative.[30] Much of the coding recorded the presence or absence of thematic content, e.g. did the article report on the GP contract or not. However, some coding required interpretation of the meaning underlying surface content, described as latent coding, [30 33] such as when recording the tone of an article. Each article's overall tone was coded as being positive, negative or neutral; this was assessed by the researcher coding that paper and discussed with the rest of the research team. Kruskal-Wallis tests were used to test the statistical significance of relationships between article tone and publication; article tone and publication genre; and variation in median article word count and publication genre. A Chi-square test was used to test differences in tone between publication genres, while a Wilcoxon signed-rank test was used to determine whether publications' media tone deviated significantly from a neutral tone. The threshold for statistical significance was set at $p<0.01$.
Statistical methods	12	(a) Describe all statistical methods, including those used to control for confounding<br>(b) Describe any methods used to examine subgroups and interactions	See point 11 for discussion of statistical analysis.<br><br>There was no missing data as the research team extracted data from all 332 articles identified in the study.

		(c) Explain how missing data were addressed<br>(d) *Cohort study* - If applicable, explain how loss to follow-up was addressed<br>*Case-control study* - If applicable, explain how matching of cases and controls was addressed<br>*Cross-sectional study* - If applicable, describe analytical methods taking account of sampling strategy<br>(e) Describe any sensitivity analyses	
Data access and cleaning methods		..	Not applicable.
Linkage		..	
Participants	13	(a) Report the numbers of individuals at each stage of the study (*e.g.*, numbers potentially eligible, examined for eligibility, confirmed eligible, included in the study, completing follow-up, and analysed)<br>(b) Give reasons for non-participation at each stage.<br>(c) Consider use of a flow diagram	There were no participants in this study.<br><br>The searches identified 332 newspaper articles for analysis.
Descriptive data	14	(a) Give characteristics of study participants (*e.g.*, demographic, clinical, social) and information on exposures and potential confounders<br>(b) Indicate the number of participants with missing data for each variable of interest<br>(c) *Cohort study* - summarise	Characteristics of the newspapers articles included, in terms of publication, publication genre, year of publication, main themes and article tone are described in the Results and in Tables 1 and 2.

For peer review only

		follow-up time (*e.g.*, average and total amount)	
Outcome data	15	*Cohort study* - Report numbers of outcome events or summary measures over time<br>*Case-control study* - Report numbers in each exposure category, or summary measures of exposure<br>*Cross-sectional study* - Report numbers of outcome events or summary measures	Not applicable.
Main results	16	(a) Give unadjusted estimates and, if applicable, confounder-adjusted estimates and their precision (e.g., 95% confidence interval). Make clear which confounders were adjusted for and why they were included<br>(b) Report category boundaries when continuous variables were categorized<br>(c) If relevant, consider translating estimates of relative risk into absolute risk for a meaningful time period	Results are expressed and number and percentage.
Other analyses	17	Report other analyses done—e.g., analyses of subgroups and interactions, and sensitivity analyses	Kruskal-Wallis tests were used to test the statistical significance of relationships between article tone and publication; article tone and publication genre; and variation in median article word count and publication genre. A Chi-square test was used to test differences in tone between publication genres, while a Wilcoxon signed-rank test was used to determine whether publications' media tone deviated significantly from a neutral tone. The threshold for statistical significance was set at $p<0.01$.
Key results	18	Summarise key results with reference to study objectives	332 newspaper articles were identified: 113 in 2005 (34.1%), 140 in 2010 (42.2%) and 79 in 2015 (23.8%). Of these, 195 (58.7%) were in quality newspapers; 99 (29.8%) in middle-market; 38 (11.3%) in tabloids. The most commonly reported themes were OOHS organisation, personal narratives and telephone triage. There was a predominance of stories about personal tragedy, including unsafe doctors and

			missed or delayed identification of rare conditions. The majority of articles (252, 75.9%) were negative in tone. This was observed for all included newspapers and by publication genre, with middle-market newspapers having the highest percentage of negative articles (Pearson Chi-Square = 35.72, p<0.001). Articles presented little supporting contextual information, such as call rates per annum, or advice on how to access OOHS.
Limitations	19	Discuss limitations of the study, taking into account sources of potential bias or imprecision. Discuss both direction and magnitude of any potential bias	Strengths and limitations are discussed on page 15, as follows:<br><br>This is the first media analysis of reporting on OOHS services. The analysis covered a comprehensive range of UK print media, including a representative spread of main publication genres, political leaning and readership demographics. The timespan covered by the analysis reflects a time of substantial change to the organisation of OOHS in the UK. The study only included newspaper articles, and there are likely to be other popular news sources excluded from this study that also exert influence on public perceptions, for example online news and social media. However, while print newspaper circulation is declining, online news is largely dominated by the online counterparts of traditional newspapers, such that analysing online news content may yield similar results.
Interpretation	20	Give a cautious overall interpretation of results considering objectives, limitations, multiplicity of analyses, results from similar studies, and other relevant evidence	The discussion on pages 12 to 15 contain out interpretation of the finding. We begin with:<br><br>This is the first study that describes UK newspaper portrayal of OOHS. Our main finding is that articles on OOHS were significantly more likely to have a negative tone than a positive or neutral tone; middle-market newspapers were significantly more likely to have a negative tone compared with the quality or tabloid newspapers. These findings highlight an over-representation of negative stories around OOHS, as illustrated by the word cloud of the most frequently occurring 'negative' headline words (Figure 1). This negativity is unrelated to newspaper political slant or readership demographics.
Generalisability	21	Discuss the generalisability (external validity) of the study results	On page 14:<br><br>This is the first media analysis of reporting on OOHS services. The analysis covered a comprehensive range of UK print media, including a representative spread of main publication genres, political leaning and readership demographics. The timespan covered by the analysis reflects a time of substantial change to the organisation of OOHS in the UK. The study only included newspaper articles, and there are likely to be other popular news sources excluded from this study that also exert influence on public perceptions, for example online news and social media. However, while print newspaper circulation is declining, online news is largely dominated by the online counterparts of traditional newspapers, such that analysing online news content may yield similar results.

For peer review only

Funding	22	Give the source of funding and the role of the funders for the present study and, if applicable, for the original study on which the present article is based	This work was unfunded.<br><br>Hamish Foster is supported by an NHS Education for Scotland Academic Fellowship. Chris Patterson's time was funded by the Informing Healthy Public Policy programme (Funded by the Medical Research Council MC_UU_12017–15 and the Chief Scientist Office SPHSU15) of the MRC/CSO Social and Public Health Sciences Unit, University of Glasgow.
Accessibility of protocol, raw data, and programming code		..	Not applicable.

For peer review only

BMJ Open

No such thing as bad publicity? A quantitative content analysis of print media representations of primary care out-of-hours services.

Journal:	*BMJ Open*
Manuscript ID	bmjopen-2018-023192.R1
Article Type:	Research
Date Submitted by the Author:	21-Nov-2018
Complete List of Authors:	Foster, Hamish; General Practice and Primary Care; University of Glasgow<br>MacDonald, Sara; University of Glasgow,<br>Patterson, Chris; University of Glasgow, MRC/CSO Social & Public Health Sciences Unit<br>O'Donnell, Catherine; University of Glasgow, General Practice & Primary Care
**Primary Subject Heading**:	General practice / Family practice
Secondary Subject Heading:	General practice / Family practice
Keywords:	PRIMARY CARE, out-of-hours, Organisation of health services < HEALTH SERVICES ADMINISTRATION & MANAGEMENT, media reporting

SCHOLARONE™
Manuscripts

No such thing as bad publicity? A quantitative content analysis of print media representations of primary care out-of-hours services.

Hamish Foster[1], Sara Macdonald[1], Chris Patterson[2], Catherine A O'Donnell[1*]

1. **General Practice & Primary Care, Institute of Health & Wellbeing, University of Glasgow.**
2. **MRC/CSO Social and Public Health Sciences Unit, Institute of Health & Wellbeing, University of Glasgow.**

Dr Hamish Foster, Clinical Academic Fellow; Hamish.Foster@glasgow.ac.uk

Dr Sara Macdonald, Senior Lecturer in Primary Care; Sara.Macdonald@glasgow.ac.uk

Mr Chris Patterson, Research Assistant; Chris.Paterson@glasgow.ac.uk

*Professor Catherine O'Donnell, Professor of Primary Care R&D; Kate.O'Donnell@glasgow.ac.uk

***Corresponding Author**

General Practice & Primary Care, Institute of Health & Wellbeing, University of Glasgow, 1 Horselethill Road, Glasgow G12 9LX. Tel: 0141 330 8329.

For peer review only

Abstract

Objective

To explore how out-of-hours primary healthcare services (OOHS) are represented in UK national newspapers, focusing on content and tone of reporting and the use of personal narratives to frame stories.

Design

A retrospective cross-sectional quantitative content analysis of articles published in 2005, 2010 and 2015.

Data Sources

Nexis database used to search 10 UK national newspapers covering quality, middle-market and tabloid publications.

Inclusion/Exclusion Criteria

All articles containing the terms "out-of-hours" [≥3 mentions per article] or ("NHS 24" OR "NHS 111" OR "NHS Direct") AND "out-of-hours" [≥1 mention per article] were included. Letters, duplicate news items, opinion pieces and articles without a substantial portion of the story (>50% of an article's wordcount, as judged by researchers) concerning OOHS were excluded.

Results

332 newspaper articles were identified: 113 in 2005 (34.1%), 140 in 2010 (42.2%) and 79 in 2015 (23.8%). Of these, 195 (58.7%) were in quality newspapers; 99 (29.8%) in middle-market; 38 (11.3%) in tabloids. The most commonly reported themes were OOHS organisation, personal narratives and telephone triage. Stories about service-level crises and personal tragedy, including unsafe doctors and missed or delayed identification of rare conditions, predominated. The majority of articles (252, 75.9%) were negative in tone. This was observed for all included newspapers and by publication genre; middle-market newspapers had the highest percentage of negative articles (Pearson Chi-

For peer review only - http://bmjopen.bmj.com/site/about/guidelines.xhtml

Square = 35.72, $p<0.001$). Articles presented little supporting contextual information, such as call rates per annum, or advice on how to access OOHS.

Conclusion

In this first reported analysis of UK national newspaper coverage of OOHS, media representation is generally negative in tone, with frequent reports of 'negative exemplars' of OOHS crises and fatal individual patient cases with little or no contextualisation. We present recommendations for the future reporting of OOHS, which could apply to the reporting of healthcare services more generally.

For peer review only

For peer review only - http://bmjopen.bmj.com/site/about/guidelines.xhtml

Strengths and Limitations

- Covers a comprehensive range of UK print media, including a representative spread of main publication genres, political leaning and readership demographics.
- Timespan covered by the analysis reflects a time of substantial change to the organisation of OOHS in the UK.
- Only newspaper articles were included.
- Other popular news sources, for example online news and social media, were excluded from this study.
- Analysis method may limit generalisability and researcher bias could not be eliminated.

For peer review only

Introduction

Primary care out-of-hours services (OOHS) is defined as primary care provided when family doctors' surgeries are closed (weekdays from 1800hr to 0800hr, weekends and public holidays) and is often delivered in community-based clinic settings. Such services generally provide care for conditions that are not life-threatening, in contrast to care delivered by hospital-based emergency departments (ED), which is available at all times and designed to manage more urgent and life-threatening problems. OOHS have seen extensive policy and structural change in many health care systems.[1-4] In many European countries, this change was characterised by a switch from small rota-based systems of primary care physicians (GPs) to regional co-operatives charged with OOHS responsibility.[5-7] In the UK, significant contractual change in 2004 offered GPs the option of transferring responsibility for OOHS to regional health organisations, leading to new models of care including out-of-hours centres, walk-in centres, and minor injuries units, as well as the implementation of national or centralised telephone triage and advice services.[1 3 8 9] Difficulties in recruiting family doctors to work in the out-of-hours period are one driver of these policy and structural changes.[4 7] A national audit of OOHS in England in 2014 found 60% of providers had gaps in their GP rotas.[10] In Scotland, older GPs typically contribute a disproportionate number of OOHS duty sessions, causing concern for future staffing of services.[11]

A second driver for change is increasing patient demand, due in part to ageing populations and associated multimorbidity,[12 13] and a concern about the supposed 'inappropriate' use of OOHS.[14 15] For example, a study of all out-of-hours calls to one region of Denmark between 2010-2011 found 24% of all out-of-hours calls were re-triaged as 'medically inappropriate' and could have been redirected to in-hours services.[16] Difficulties accessing daytime primary care have also been reported as leading to increased use of OOHS.[17 18] Patients have also reported uncertainty about

For peer review only - http://bmjopen.bmj.com/site/about/guidelines.xhtml

For peer review only

the urgency of presenting complaints, limited knowledge about when and how to access OOHS and confusion about which services to access, all of which may drive them towards using more visible and accessible emergency departments, rather than primary care OOHS.[19 20] Thus, patients need to be able to access information that helps them make decisions about where, and how, to access health care, especially for more urgent issues.

Print media remains a common source of public knowledge and a potentially powerful influence on peoples' perceptions. Newspapers are able to set public agendas, determining *what* issues are of public importance.[21] Newspapers also 'frame' issues, influencing *how* topics are viewed.[22] Negative newspaper coverage of an issue is not only associated with negative perceptions,[23] but with negative health behaviour outcomes, such as lower vaccine uptake.[24] Equally concerning are the numerous empirical examples of the misrepresentation of health issues by print media. For example, newspaper articles have been found to be overly positive in the reporting of new surgical interventions, to under-report epidemiological data in relation to the HPV vaccine,[25 26] and to report stories about rare diseases more frequently than common conditions.[27] Media analyses in the UK have also suggested that negative newspaper portrayals of GPs, and GP pay, are linked to decreasing professional morale and GP recruitment difficulties.[28 29] However, to our knowledge, there has been no previous examination of how UK or international newspapers portray OOHS despite the aforementioned significant policy and structural changes. Improved understanding of this important influence on public, and staff, perceptions may inform OOHS service providers in their patient education campaigns and in staff recruitment drives.

This study aimed to explore how UK OOHS are portrayed in national newspapers, with a particular focus on the content and tone of reporting and the use of personal narratives or individual case studies to frame stories.

For peer review only - http://bmjopen.bmj.com/site/about/guidelines.xhtml

Methods

Selection of national newspapers

Ten UK national newspapers were purposively selected to represent the breadth of UK national print media, in terms of newspaper genre (quality, middle-market or tabloid), current political alignment and readership demographics.[30-32] Newspapers included major UK and Scottish titles, and their Sunday equivalents, and comprised: *Telegraph*, *Times* and *Guardian* (UK quality publications); the *Herald* and the *Scotsman* (Scottish quality publications); the *Daily Mail* and the *Daily Express* (UK middle-market publications); the *Daily Mirror* and the *Daily Star* (UK tabloids) and the *Daily Record* (Scottish tabloid). Full details are presented in Appendix 1. Scottish titles were considered separately as, in the UK, health is a devolved matter and under the jurisdiction of the Scottish Government, with well-recognised variation in both health strategy and the organisation of service delivery.[33 34]

Searches

Searches were conducted using the Nexis database; the chosen timeframe was 1st January 2004 until 31st October 2015, which incorporated the implementation of the new General Medical Services (GMS) contract for general practitioners in the UK, with the changes to OOHS provision as previously described.[1 35] All articles containing the terms "out-of-hours" [≥3 mentions per article] or ("NHS 24" OR "NHS 111" OR "NHS Direct" (the names of UK NHS telephone triage and advice services)) AND "out-of-hours" [≥1 mention per article] were included. Letters, duplicate news items, opinion pieces without editorial content and articles without a substantial portion of the story (>50% of an article's word count, as judged by researchers) concerning OOHS were excluded. The searches returned 1625 articles in total; we purposively selected all articles published in the years 2005, 2010 and 2015 for detailed content analysis, in order to provide a snapshot of reporting over this timeframe.

For peer review only - http://bmjopen.bmj.com/site/about/guidelines.xhtml

Coding and analysis

A retrospective cross-sectional quantitative content analysis was conducted using a coding pro forma developed iteratively by all the authors.[32 36 37] The pro forma recorded how often, and in which newspapers, stories about OOHS were reported; the type of story (news item, feature article, or editorial); and the main theme of the article. Main themes were identified by the study team as those relevant to the research aims, including: OOHS organisation; demand/volume of work; GP contract; and personal narrative or case study. Most of the themes were identified by the team prior to coding and were based on a priori knowledge of OOHS provision and research.[38] Further themes were identified inductively as they emerged during the coding process. When a personal narrative was reported, demographic details about the patient, the clinical problem presented to the OOHS and the outcome were recorded. Articles could be coded as having more than one theme, for example service organisation and a personal narrative.

HF, SM and CO'D read and coded a total of 100 articles using the pro forma. The first 30 articles in each year were triple coded by these authors. All authors discussed this coding and designed the pro forma, adding and refining themes and recorded content as identified (e.g. adding themes related to whether articles used supporting statistics or gave advice to readers about accessing OOHS). Following that, the same authors each selected one identified year (2005, 2010 or 2015) and coded the remaining articles from that year. Coding decisions were discussed within the team throughout this process to ensure consistency.

Data were entered into STATA for statistical analysis. Textual data on the themes contained in the articles were coded numerically (1 = yes, theme was reported; 2 = no, theme was not reported); thus, the resulting analysis was quantitative.[32] Much of the coding recorded the presence or absence of thematic content, e.g. did the article report on the GP contract or not. However, some coding

For peer review only - http://bmjopen.bmj.com/site/about/guidelines.xhtml

required interpretation of the meaning underlying surface content, described as latent coding, [32 36] such as when recording the tone of an article. Each article's overall tone was coded as being positive, negative or neutral; this was assessed by the researcher coding that paper and discussed with the rest of the research team. Kruskal-Wallis tests were used to test the statistical significance of relationships between article tone and publication; article tone and publication genre; and how the median word count of articles varied by publication genre. A Chi-square test was used to test differences in tone between publication genres, while a Wilcoxon signed-rank test was used to determine whether publications' media tone deviated significantly from a neutral tone. The threshold for statistical significance was set at $p<0.01$.

Patient Involvement

The stimulus for this study came from work conducted for the Scottish Government's Out-of-Hours Review Group, which included a range of policy, professional and patient stakeholder groups. However, patients were not explicitly involved in the design or interpretation of the work reported here.

Results

There were 332 articles from the sampled years that met the inclusion criteria: 113 (34.1%) published in 2005; 140 (42.2%) in 2010; and 79 (23.8%) in 2015. Overall 182 (54.8%) articles were news reports; 44 (13.3%) were features; and 99 (29.8%) were editorials. Of the 332 articles, 195 (58.7%) were in the quality press; 99 (29.8%) in middle-market newspapers; and 38 (11.5%) in the tabloids. While there was variation across publications and by year, the *Daily Mail/Mail on Sunday* published the most articles (n=76 (22.9% of total)) whereas the *Daily Star/Daily Star Sunday* published just one

For peer review only - http://bmjopen.bmj.com/site/about/guidelines.xhtml

article (Figure 1). The *Guardian/Observer* and *Daily Telegraph/Sunday Telegraph* both published substantially more articles on OOHS in 2010 than in either 2005 or 2015, though it should be noted that the last two months of 2015 were not accounted for in the sample.

The overall median word count was 508.0 words (25th to 75th percentiles: 323.0 to 687.5). Median word count was highest in the middle-market newspapers and lowest in the tabloids (Quality: median 491.0 (25th to 75th percentiles: 329.0 to 654.0); Middle-market: median 612.0 (25th to 75th percentiles: 445.0 to 804.0; Tabloid: median 336.5 (25th to 75th percentiles: 199.3 to 504.0) (Kruskall-Wallis $p<0.001$).

Thematic content

Thematic content was coded 621 times across the 332 articles, thus most articles reported more than one theme. The most frequently reported themes were service organisation, personal narratives, and telephone triage (Table 1), but there were variations by year and publication genre.

Service organisation was the most frequent theme because all other themes or stories within articles were frequently discussed with respect to wider organisational or structural issues. For example, articles focussing on personal narratives would often relate the narrative to staff shortages or OOHS organisational changes. Other subthemes within service organisation included discussion around models of care (e.g. commercial agencies' provision of OOHS), IT systems, and political discourse on OOHS organisation. Articles reporting personal narratives were second most frequent and were particularly frequent in 2010 (n=83), prompting closer examination of these articles (see below). In 2005, telephone triage was a recurrent theme; 53 of 60 references to telephone triage in 2005 related to a widely publicised report on NHS 24, the then relatively new Scottish telephone triage service, following a fatal accident inquiry.[39] The theme of telephone triage in 2005 also linked to the service organisation theme, because articles reporting telephone triage often discussed wider

For peer review only - http://bmjopen.bmj.com/site/about/guidelines.xhtml

For peer review only

OOHS organisational issues. Broadly similar frequencies of topics were seen across the three publication genres, but tabloid newspapers published fewer articles about service organisation.

Articles coded as 'other' encompassed a range of topics, including service response times; patient satisfaction; GP OOH responsibility; confusion around services; and quality of care. In each case, there were only 1 to 5 articles concerned with each topic. The exception was the theme of 'unsafe non-UK doctors', which was a recurrent theme in 2010, comprising 58 of the 83 articles coded as 'other'. Inspection of these 58 articles found that 53 (91.4%) of these occurrences were in newspaper articles also coded to the theme of 'personal narrative'. This is presented in more detail in the section entitled Reporting of personal narratives.

As well as recording main themes of articles, mentions of specific issues were recorded as part of the pro forma. Twelve (3.6%) OOHS news articles mentioned explicit self-management advice for patients or offered information on how to use OOHS appropriately, and only 1 article contained advice on accessing OOHS. Conversely, 26 articles (7.8%) described confusion around OOHS. Numerical data (e.g. focused on reduced numbers of staff on duty, large number of calls or home visits delayed, high annual costs) were frequently cited in articles but denominator or contextual data was rarely given alongside the headline statistic, for example the call rate per annum or number of cases of meningitis seen by an OOHS in one year.

Article Tone

Overall, 252 of the 332 articles (75.9%) were negative with only 11 (3.3%) positive, and 69 (20.1%) neutral (Table 2). Articles published in the middle-market newspapers were more likely to be negative in tone (86/99; 86.9%) compared with the quality newspapers (136/195; 69.7%) or the tabloids (30/38; 79.0%) (Pearson Chi-Square = 35.72, $p<0.001$). Each individual publication leaned significantly toward a negative tone (Wilcoxon signed-rank $p<0.01$). While no publication was

For peer review only - http://bmjopen.bmj.com/site/about/guidelines.xhtml

For peer review only

significantly more positive/negative/neutral than any other (Kruskall-Wallis p=0.025) some – notably the Scottish quality newspapers – tended towards a more neutral tone of reporting (Table 2). Tone also varied by year, with 69.9%, 90.7% and 58.2% of articles being negative in tone for years 2005, 2010, and 2015 respectively.

Reporting of personal narratives

Nearly half of the articles (153; 46.1%) mentioned at least one personal narrative, with the majority portraying such stories as tragedies (132/153; 86.3%). Most featured the personal narrative as the main theme (108/153; 70.6%); however, others commented on a personal narrative in passing, for example at the end of an article. Where personal narrative was a main theme, the majority described narratives that involved rare diagnoses or problems (104/108; 96.3%). Examples included fatal iatrogenic overdose, fatal sepsis or fatal meningitis in younger patients. Individual cases were also repeated in multiple stories. This was particularly apparent in one story, reported in 2010, of an overseas doctor working in an OOHS who accidently administered a fatal overdose of diamorphine to a patient during the doctor's first OOHS shift in the UK. Of the 140 articles on OOHS published in 2010, 89 (63.6%) referenced this case. Of these 89 articles, 58 (65.2%) were coded to the theme of 'unsafe non-UK doctors' – meaning that a main theme of these articles was this story. The remaining 31 articles used this story indirectly to illustrate a point in an unrelated article.

Discussion

This is the first study that describes UK newspaper portrayal of OOHS. Our main finding is that articles on OOHS were significantly more likely to have a negative tone than a positive or neutral tone; middle-market newspapers were significantly more likely to have a negative tone compared with the

For peer review only - http://bmjopen.bmj.com/site/about/guidelines.xhtml

quality or tabloid newspapers. This negativity was unrelated to newspaper political slant or readership demographics. These findings highlight an over-representation of negative stories around OOHS, as illustrated by the word cloud of the most frequently occurring 'negative' headline words (Figure 2). This figure was created by collating all the negative headline terms whereby the size of the word was determined by the frequency that the word appeared in different headlines, with larger words representing more frequently used words. This negative representation of OOHS contrasts with patient views of care, with the 2017 GP patient survey in England finding that 66.2% of respondents who had recently accessed OOHS rated their overall experience as 'fairly or very good'.[40] There has, however, been a slight drop in satisfaction since the question was first asked in the 2012 survey, where 70.9% of respondents replied positively.[41] While this slight decrease cannot be attributed to media coverage, it appears part of a pattern of increasingly negative attitudes towards primary care services both in terms of patient satisfaction and in terms of media reporting. Two previous studies of GP portrayal and GP pay identified a change in GP newspaper portrayal over time related to contractual change, with portrayals of both GPs and their salaries becoming more negative after the introduction of the 2004 GMS contract.[28 29] However, our study found much higher levels of negativity, in terms of reporting, than either of the two previous studies. This may have implications for patients' on-going trust of both OOHS, and GPs in general.

Our results illustrate how a single issue within the OOHS can characterise a large proportion of OOHS related news. For example, telephone triage services were a dominant theme in 2005, with many articles related to a report published by the Scottish Government on the Scottish telephone triage service, NHS 24.[42] This report was often referred to in the media when reporting on a judicial inquiry into the deaths of a teenager from meningitis and a 30 year old man from sepsis, both of whom had contacted the triage service prior to their deaths, or in relation to patient complaints of

For peer review only - http://bmjopen.bmj.com/site/about/guidelines.xhtml

long ‘call-back’ waiting times for NHS24. This is consistent with previous media analysis that have highlighted a UK media focus on health service ‘crises’,[28 29] or on more negative aspects of public health programmes, such as perceived adverse impacts of vaccination.[43 44] This suggests that negative news reports on OOHS are part of a wider and persistent pattern of negative print media reporting about the NHS.

Our analysis also identified the important role that a single case can play in framing media representation of OOHS. Some cases will indeed be worth reporting and a missed diagnosis resulting in a death is always a tragic incident. However, such incidents are rare and persistent or repeated reporting of particular cases could provide a skewed picture of OOHS. For example, the reporting in 2010 of the Coroner’s ruling on the unlawful killing of a patient by a doctor working in an OOHS dominated the reporting that year. Although an incomplete calendar year was included for 2015, there appeared to be more OOHS articles published in 2010 than in the other two years, and a large proportion of them reported on this case either directly or indirectly. In addition, a higher proportion of articles published in 2010 were negative, compared with the other two years sampled. This case was often included in unrelated articles as an exemplar of an OOHS tragedy. This use of ‘negative exemplars’ was also seen in relation to personal narratives about the presentation of rare clinical problems, such as septicaemia or meningitis, with such cases often used in passing at the end of unrelated OOHS articles. This is in line with previous media analysis that showed leading US magazines and newspapers over-represented infrequent causes of death while under-representing common causes.[27] Similarly, a study of UK media showed the number of ‘deaths-per-news-story’ was much higher for common causes (e.g. smoking) of death compared with rare causes (e.g. vCJD).[43] Public perception of the safety of OOHS could therefore be skewed by an over-

For peer review only

For peer review only - http://bmjopen.bmj.com/site/about/guidelines.xhtml

representation of rare and tragic cases. This is coupled with low levels of reliable statistical reporting and with very few articles offering advice on when, and how, to access OOHS.

While the media have an important role in holding public bodies and services to account, we believe the media also has a responsibility to portray public services fairly and to provide related impartial information. Misrepresentations may affect people's interaction with, and outcomes from, OOHS either by increasing demand through unnecessary concern over rare illnesses or, alternatively, through eroding public trust and therefore delaying use of the service. Doctors may also be reluctant to work in OOHS where they may be concerned about managing risk in undifferentiated presentations of acute illness and the possible consequences if they are judged to 'make a mistake'.

Despite a preponderance of reporting on crises within and confusion around OOHS services, newspapers offered little practical guidance on accessing care. Thus, to counteract skewed representations of OOHS and their staff, to reduce inappropriate OOHS use and to improve public understanding of health services we recommend the development of guidelines for media reporting on health services. While the starting point here is OOHS, these could be adapted to other healthcare settings.

Developing reporting guidelines for OOHS

National and international guidelines already exist for the media reporting of suicides,[45 46] with the threat of failure to adhere to such guidelines in the UK resulting in referral to the UK Press Complaints Commission. Best practice guidelines also exist for UK media reporting of scientific studies.[47] However, these guidelines focus on research findings and omit recommendations specific to health services that are of public health importance. Box 1 contains suggested recommendations on OOHS reporting that could be integrated into current reporting guidelines.

For peer review only

Although these guidelines are in response to an analysis of UK print media they are likely to be relevant in countries with publicly funded healthcare systems and similar media reporting.

Box 1. Media reporting guidelines for articles reporting on OOHS.

1. Media reporting of OOHS should avoid the practice of adding a 'negative exemplar' at the end of a story unrelated to that personal narrative.
2. Media sources are encouraged to consider adopting the practice of 'positive exemplars', namely personal narratives where someone received timely and appropriate care, perhaps for a rare or unusual clinical problem.
3. Where personal narratives are reported to depict poor care and specific diseases or illnesses are mentioned, provide further disease-related information or direct readers to further information from providers of impartial health advice. For example, on reporting following a case of fatal sepsis in a young person direct the reader to the website of a prominent sepsis charity/organisation.
4. When reporting on service access problems direct readers to further information on how and when to navigate services. For example, if reporting on a case of delay in care direct the reader to NHS Choices or provide the local telephone triage service number.
5. When reporting health service related statistics provide contextual data. For example, if reporting on how few doctors are covering an OOHS shift, provide information or provide links to information on how many staff normally cover that shift. Or if reporting on costs of a service direct readers to more information on costs of other public services.
6. When reporting on use of an OOHS, provide contextual information such as the number of calls or visits the service received per annum.
7. Where possible, provide links to local information or campaigns about alternative sources of health care support e.g. local pharmacies.
8. OOHS health professionals, managers and service designers should increase, enhance and capitalise on opportunities to work with journalists and editors to help publishers follow these recommendations.

Strengths and limitations

This is the first media analysis of reporting on OOHS services. The analysis covered a comprehensive range of UK print media, including a representative spread of main publication genres, political leaning and readership demographics. The timespan covered by the analysis reflects a time of

For peer review only - http://bmjopen.bmj.com/site/about/guidelines.xhtml

substantial change to the organisation of OOHS in the UK. The study only included newspaper articles, and there are likely to be other popular news sources excluded from this study that also exert influence on public perceptions, for example online news and social media. However, while print newspaper circulation is declining, online news is largely dominated by the online counterparts of traditional newspapers, such that analysing online news content may yield similar results. Examining related social media data was beyond the scope of this study but future research in this area would be valuable given the rise in social media use. The combination of deductive and inductive approaches to collect data based on both priori themes and emergent themes respectively may affect the reproducibility and therefore generalisability of our findings. Similar analyses in other settings, including international settings, are required. Finally, we cannot rule out individual researcher bias as most articles were coded by a single researcher. However, this bias might be limited as approximately one third of the articles were independently coded by three researchers after which discrepancies were discussed. Consensus was easily reached before the remaining articles were coded.

Conclusion

In summary, we found UK newspaper OOHS reporting to be generally negative in tone, irrespective of newspaper type, which, in keeping with previous media analyses, included a preponderance of articles describing crises or personal narratives depicting rare and tragic patient stories. Uniquely, we found that media reports related to OOHS can become dominated by a single personal narrative. Our findings provide clear examples of media representations that may negatively affect the public's perceptions of, and interaction with, OOHS. Developing guidelines to encourage responsible reporting on health services may have a role in reducing the risk of skewed public perceptions.

For peer review only

Further research that examines public perceptions of OOHS in light of these newspaper representations would develop understanding of the media’s role in shaping public opinion of health services.

Contributors.

HF, SM and COD conceived the idea and designed the study; HF and CP designed the search strategies, with input from SM and COD. All authors contributed to the design of the data extraction pro forma and data extraction. HF and CP analysed the data; all authors contributed to data interpretation. HF wrote the first draft; COD led the re-drafting; all authors contributed to the final version and agreed to its submission. COD is the guarantor.

Funding.

This study was unfunded. HF is supported by an NHS Education for Scotland Academic Fellowship. CP’s time was funded by the Informing Healthy Public Policy programme (Funded by the Medical Research Council MC_UU_12017–15 and the Chief Scientist Office SPHSU15) of the MRC/CSO Social and Public Health Sciences Unit, University of Glasgow.

Competing interests.

All authors have completed the ICMJE uniform disclosure form at http://www.icmje.org/coi_disclosure.pdf and declare: no support from any organisation for the submitted work; no financial relationships with any organisations that might have an interest in the submitted work in the previous three years, no other relationships or activities that could appear to have influenced the submitted work.

Ethical approval.

For peer review only

For peer review only - http://bmjopen.bmj.com/site/about/guidelines.xhtml

Ethical approval was not required for this study.

Data sharing.

Copies of the search strategy, identified media articles and data extraction pro forma are available on request to HF or COD.

Copyright/License for publication.

The Corresponding Author has the right to grant on behalf of all authors and does grant on behalf of all authors, a worldwide licence to the Publishers and its licensees in perpetuity, in all forms, formats and media (whether known now or created in the future), to i) publish, reproduce, distribute, display and store the Contribution, ii) translate the Contribution into other languages, create adaptations, reprints, include within collections and create summaries, extracts and/or, abstracts of the Contribution, iii) create any other derivative work(s) based on the Contribution, iv) to exploit all subsidiary rights in the Contribution, v) the inclusion of electronic links from the Contribution to third party material where-ever it may be located; and, vi) licence any third party to do any or all of the above.

For peer review only - http://bmjopen.bmj.com/site/about/guidelines.xhtml

References

1. Hurwitz B. The new out of hours agreement for general practitioners. *BMJ* 1995;311:824-25.

2. Hansen BL, Munck A. Out-of-hours service in Denmark: The effect of a structural change. *British Journal of General Practice* 1998;48:1497-99.

3. Tan S, Mays N. Impact of initiatives to improve access to, and choice of, primary and urgent care in the England: A systematic review. *Health Policy* 2014;118:304-15.

4. Smits M, Rutten M, Keizer E, et al. The development and performance of after-hours primary care in the Netherlands: A narrative review. *Annals of Internal Medicine* 2017;166:737-42 doi: 10.7326/M16-2776.

5. Christensen MB, Olesen F. Out of hours service in Denmark: evaluation five years after reform. *BMJ* 1998;316:1502-05.

6. van Uden CJT, Giesen PHJ, Metsemakers JFM, et al. Development of out-of-hours primary care by general practitioners (GPs) in The Netherlands: from small-call rotations to large-scale GP cooperatives. Family Medicine 2006;**3**8:565-69.

7. Berchet C, Nader C. The organisation of out-of-hours primary care in OECD countries: OECD Publishing, 2016.

8. Munro JF, Nicholl JP, O'Cathain A, et al. Impact of NHS Direct on demand for immediate care: observational study. *BMJ* 2000;321:150-53.

9. Turner J, O'Cathain A, Knowles E, et al. Impact of the urgent care telephone service NHS 111 pilot sites: a controlled before and after study. *BMJ Open* 2013;3:e003451.

10. National Audit Office. Out-of-hours GP services in England, 2014.

For peer review only

For peer review only - http://bmjopen.bmj.com/site/about/guidelines.xhtml

11. The Scottish Government. Pulling together: transforming urgent care for the people of Scotland. The Report of the Independent Review of Primary Care Out of Hours Services: National Review of Primary Care Out of Hours Services. A Scottish Government commisioned report, 2015.

12. Barnett K, Mercer SW, Norbury M, et al. Epidemiology of multimorbidity and implications for health care, research, and medical education: a cross-sectional study. *The Lancet* 2012;380:37-43 doi: 10.1016/S0140-6736(12)60240-2.

13. Hobbs FDR, Bankhead C, Mukhtar T, et al. Clinical workload in UK primary care: a retrospective analysis of 100 million consultations in England, 2007-14. *The Lancet* 2016;387:2323-30 doi: 10.1016/S0140-6736(16)00620-6.

14. Kelly SJ, Piercy H, Ibbotson R, et al. Who attends out-of-hours general practice appointments? Analysis of a patient cohort accessing new out-of-hours units. *BMJ Open* 2018;8:e020308 doi: 10.1136/bmjopen-2017-020308.

15. Keizer E, Smits M, Peters Y, et al. Contacts with out-of-hours primary care for nonurgent problems: patients' beliefs or deficiencies in healthcare? *BMC Fam Pract* 2015;16:157 doi: 10.1186/s12875-015-0376-9.

16. Noroxe KB, Huibers L, Moth G, et al. Medical appropriateness of adult calls to Danish out-of-hours primary care: a questionnaire-based survey. *BMC Fam Pract* 2017;18:34 doi: 10.1186/s12875-017-0617-1.

17. Zhou Y, Abel G, Warren F, et al. Do difficulties in accessing in-hours primary care predict higher use of out-of-hours GP services? Evidence from an English National Patient Survey. *Emergency Medicine Journal* 2015;32:373-78.

18. Smits M, Peters Y, Broers S, et al. Association between general practice characteristics and use of out-of-hours GP cooperatives. *BMC Family Practice* 2015;16:52.

For peer review only - http://bmjopen.bmj.com/site/about/guidelines.xhtml

19. MacKichan F, Brangan E, Wye L, et al. Why do patients seek primary medical care in emergency departments? An ethnographic exploration of access to general practice. *BMJ Open* 2017;7 doi: 10.1136/bmjopen-2016-013816.

20. Weber EJ, Hirst E, Marsh M. The patient’s dilemma: attending the emergency department with a minor illness. *BMJ* 2017;357:j1941 doi: 10.1136/bmj.j1941.

21. Shaw DL, Martin SE. The Function of mass-media agenda setting. *Journalism Quart* 1992;69(4):902-20.

22. Scheufele DA. Framing as a theory of media effects. *J Commun* 1999;49:103-22 doi: DOI 10.1111/j.1460-2466.1999.tb02784.x.

23. Nagelhout GE, van den Putte B, de Vries H, et al. The influence of newspaper coverage and a media campaign on smokers' support for smoke-free bars and restaurants and on secondhand smoke harm awareness: findings from the International Tobacco Control (ITC) Netherlands Survey. *Tobacco Control* 2012;21:24-9 doi: 10.1136/tc.2010.040477.

24. Mason BW, Donnelly PD. Impact of a local newspaper campaign on the uptake of the measles mumps and rubella vaccine. *Journal of Epidemiology and Community Health* 2000;54:473-74 doi: DOI 10.1136/jech.54.6.473.

25. Malviya A, Stafford GH, Villar RJF, et al. Have the media influenced the use of hip resurfacing arthroplasty? A review of UK print media. *Ann Roy Coll Surg* 2012;94:432-37 doi: 10.1308/003588412x13171221592014.

26. Bodemer N, Müller SM, Okan Y, et al. Do the media provide transparent health information? A cross-cultural comparison of public information about the HPV vaccine. *Vaccine* 2012;30:3747-56 doi: https://doi.org/10.1016/j.vaccine.2012.03.005.

27. Frost K, Frank E, Maibach E. Relative risk in the news media: A quantification of misrepresentation. *Am J Public Health* 1997;87:842-45 doi: Doi 10.2105/Ajph.87.5.842[published Online First: Epub Date]|.

For peer review only

For peer review only - http://bmjopen.bmj.com/site/about/guidelines.xhtml

28. Balkham A, Alderson S. 'The biggest car crash in NHS history': the media portrayal of GP pay before and after the introduction of the Health and Social Care Bill 2011. *Primary Health Care Research & Development* 2017;18:84-91 doi: 10.1017/S1463423616000359.

29. Tanner F, Foy R, Harrison W. 'Wads up, Doc' - trends in British newspapers' reporting of general practitioners' pay. *Primary Health Care Research & Development* 2010;11:405-09

30. Hilton S, Patterson C, Teyhan A. Escalating coverage of obesity in UK newspapers: The evolution and framing of the "obesity epidemic" from 1996 to 2010. *Obesity* 2012;20:1688-95 doi: 10.1038/oby.2012.27.

31. Rooke C, Amos A. News media representations of electronic cigarettes: an analysis of newspaper coverage in the UK and Scotland. *Tobacco Control* 2014;23:507-12 doi: 10.1136/tobaccocontrol-2013-051043.

32. Patterson C, Katikireddi SV, Wood K, et al. Representations of minimum unit pricing for alcohol in UK newspapers: a case study of a public health policy debate. *Journal of Public Health* 2015;37:40-49 doi: 10.1093/pubmed/fdu078.

33. Bevan G, Karanikolos M, Exley J, et al. The four health systems of the United Kingdom: how do they compare? London, 2014.

34. Greer SL. Devolution and health in the UK: policy and its lessons since 1998. *British Medical Bulletin* 2016;118:16-24 doi: 10.1093/bmb/ldw013.

35. Roland M. Linking physicians' pay to the quality of care--a major experiment in the United kingdom. *N Engl J Med* 2004;**351**:1448-54 doi: 10.1056/NEJMhpr041294.

36. Downe-Wamboldt B. Content analysis: method, applications, and issues. *Health Care for Women International* 1992;**13**:313-21 doi: 10.1080/07399339209516006.

37. Manganello J, Blake N. A study of quantitative content analysis of health messages in U.S. media from 1985 to 2005. *Health Communication* 2010;25(5):387-96 doi: 10.1080/10410236.2010.483333.

For peer review only

For peer review only - http://bmjopen.bmj.com/site/about/guidelines.xhtml

38. O'Donnell CA, Foster H, Macdonald S, et al. Out-of-hours primary medical care: What can research tell us? Findings from a rapid systematic review and qualitative study. Scottish Government: General Practice & Primary Care, Institute of Health and Wellbeing, University of Glasgow, 2015.

39. Scottish Courts and Tribunals. Fatal accident inquiry into the deaths of Shomi Moshina and Steven Alexander Wiseman. Secondary Fatal accident inquiry into the deaths of Shomi Moshina and Steven Alexander Wiseman 2006. https://www.scotcourts.gov.uk/search-judgments/judgment?id=be3d87a6-8980-69d2-b500-ff0000d74aa7.

40. Ipsos MORI, NHS England. GP patient survey 2017. National results and trends: NHS England, 2017.

41. Ipsos MORI, England N. The GP patient survey 2011/2012. Summary report, 2012.

42. NHS 24 Independent Review Team. Review of NHS 24. Final Report, 2005.

43. Harrabin R, Coote A, Allen J. Health in the news. Risk, reporting and media influence. King's Fund Publications: King's Fund, 2003.

44. Hilton S, Hunt K, Langan M, et al. Newsprint media representations of the introduction of the HPV vaccination programme for cervical cancer prevention in the UK (2005–2008). *Social Science & Medicine* 2010;70:942-50 doi:.

45. The Samaritans. Media guidelines for reporting suicide: The Samaritans, 2017.

46. World Health Organization, International Association for Suicide Prevention. Preventing suicide. A resource for media professionals, 2008.

47. Science Media Centre. 10 best practice guidelines for reporting science and health stories: Science Media Centre, 2012.

For peer review only - http://bmjopen.bmj.com/site/about/guidelines.xhtml

For peer review only

For peer review only - http://bmjopen.bmj.com/site/about/guidelines.xhtml

Table 1. Reporting of OOHS themes by year and by publication genre (Number of times theme coded (%))*

Theme	Total number of times theme coded (n = 621)	Publication year (n, %)			Publication genre (n, %)		
		2005 (n = 174)	2010 (n = 332)	2015 (n = 115)	Quality (n = 365)	Middle-market (n = 192)	Tabloid (n = 64)
Service organisation	139	40 (23.0)	75 (22.6)	24 (20.9)	92 (29.5)	40 (25.8)	7 (10.9)
Case study/Personal narrative	108	13 (7.5)	83 (25.0)	12 (10.4)	62 (19.9)	33 (21.3)	13 (20.3)
Telephone triage	95	60 (34.3)	11 (3.3)	24 (20.9)	59 (18.9)	21 (13.5)	15 (23.4)
GP contract	52	18 (10.3)	30 (9.0)	4 (3.5)	25 (8.0)	25 (16.1)	2 (3.1)
Demand/Volume of work	44	16 (9.2)	10 (3.0)	18 (15.7)	28 (9.0)	12 (7.7)	4 (6.2)
GP pay	33	12 (6.9)	17 (5.1)	4 (3.5)	15 (4.8)	13 (8.5)	5 (7.8)
Rurality	20	3 (1.7)	15 (4.5)	2 (1.7)	17 (5.4)	0 (0.0)	3 (4.7)
Seven day services	12	2 (1.2)	1 (0.3)	9 (7.8)	4 (1.3)	7 (4.5)	1 (1.6)
Public responsibility/Appropriateness	10	1 (0.6)	3 (0.9)	6 (5.2)	4 (1.3)	2 (1.3)	4 (6.2)
Cost of services	8	2 (1.2)	4 (1.3)	2 (1.7)	6 (1.9)	2 (1.3)	0 (0.0)
Other	100	7 (4.0)	83 (25.0)	10 (8.7)	53 (17.0)	37 (23.9)	10 (15.7)

*Articles coded to more than 1 theme

For peer review only - http://bmjopen.bmj.com/site/about/guidelines.xhtml

Table 2. Tone by newspaper publication (Number of articles, (% within newspaper publication))

Publication	Positive tone (n=11)	Negative tone (n=252)	Neutral tone (n=69)
**Quality**			
Herald/Sunday Herald (n=51)	1 (2.0)	30 (58.8)	20 (39.2)
Times/Sunday Times (n=40)	0 (0.0)	32 (80.0)	8 (20.0)
Daily Telegraph/Sunday Telegraph (n=35)	1 (2.9)	26 (74.3)	8 (22.8)
Guardian/Observer (n=38)	2 (5.3)	29 (76.3)	7 (18.4)
Scotsman/Scotland on Sunday (n=31)	1 (3.2)	19 (61.3)	11 (35.5)
**Middle-Market**			
Daily Mail/Mail on Sunday (n=76)	0 (0.0)	67 (88.2)	9 (11.8)
Express/Sunday Express (n=23)	0 (0.0)	19 (82.6)	4 (17.4)
**Tabloid**			
Daily Record/Sunday Mail (n=25)	5 (20.0)	19 (76.0)	1 (4.0)
Daily Mirror/Sunday Mirror (n=12)	1 (8.3)	10 (83.3)	1 (8.3)
Daily Star/Daily Star Sunday (n=1)	0 (0.0)	1 (100.0)	0 (0.0)

For peer review only

Figures.

Figure 1. Article frequency by publication and year.

Figure 2. Word cloud of most frequently appearing negative words in headlines about OOHS services.

For peer review only

For peer review only - http://bmjopen.bmj.com/site/about/guidelines.xhtml

Figure 1. Article frequency by publication and year.

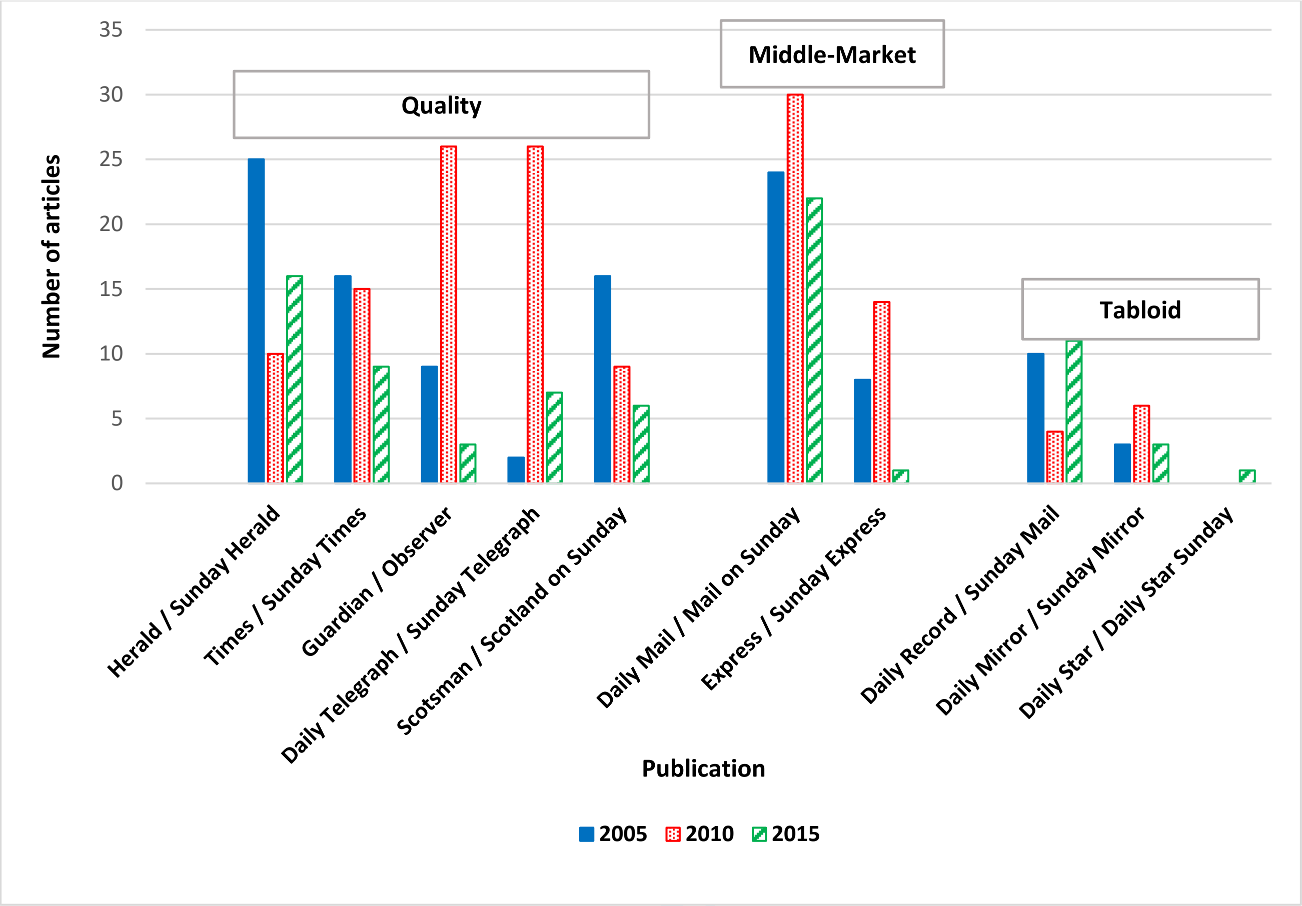

Figure 2. Word cloud of most frequently appearing negative words in headlines about OOHS services.

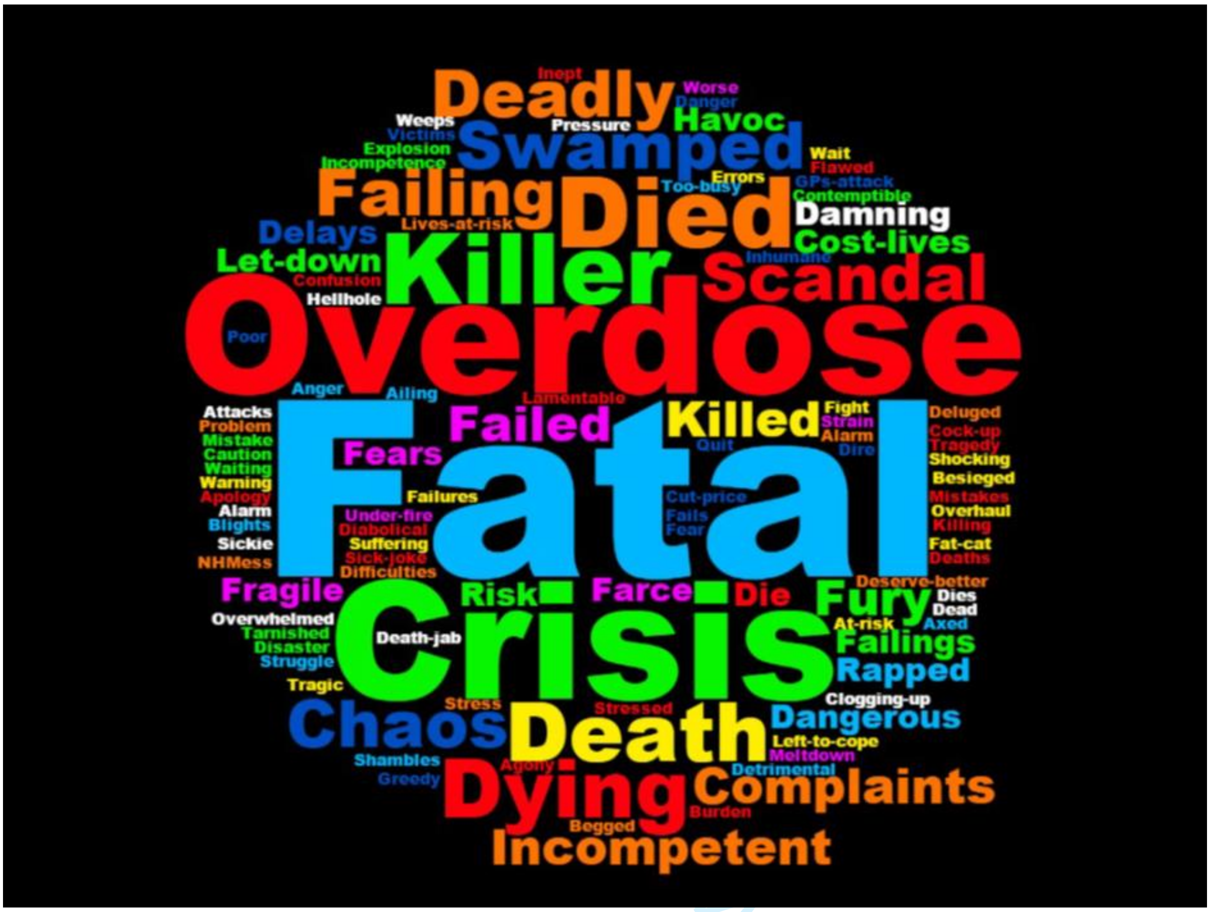

Appendix 1. Overview of main UK and Scottish daily and Sunday newspapers selected by publication location, genre, circulation, readership and political ideology, October 205 – September 2016. (From: National Readership Survey, http://www.nrs.co.uk/downloads/pdf/newspapers_201611.pdf)

Newspaper	Estimated weekly readership (Number)	% Readership Female	% Readership in Social Group C2DE	% Readership Aged 35 +	Political Ideology
**UK**					
**Quality**					
Daily Telegraph	1,183,000	47.4	17.8	89.6	Right
The Sunday Telegraph	1,113,000	46.9	15.5	89.2	Right
The Times	1,047,000	44.6	15.2	83.8	Centre-right
The Sunday Times	1,834,000	47.0	15.4	83.9	Centre-right
The Guardian	865,000	48.4	20.1	73.9	Centre-left
The Observer	764,000	45.8	13.2	80.9	Centre-left
**Middle-Market**					
Daily Mail	3,354,000	53.9	38.0	87.7	Right
The Mail on Sunday	3,608,000	51.5	39.2	87.0	Right
Daily Express	842,000	48.8	43.6	91.8	Right
Sunday Express	826,000	47.5	40.4	88.0	Right
**Tabloid**					
The Sun*	4,188,000	42.7	68.3	75.5	Right
The Sun on Sunday*	3,640,000	44.6	65.3	72.4	Right
Daily Mirror	1,818,000	46.1	65.0	83.7	Left
Sunday Mirror	1,804,000	49.8	61.5	79.0	Left
Daily Star	800,000	34.5	75.1	74.8	Neutral
Daily Star Sunday	584,000	40.8	74.5	71.2	Neutral

For peer review only

**Scotland**					
**Quality**					
The Herald	109,000	33.0	26.6	87.2	Neutral
Sunday Herald	109,000	46.8	30.3	73.4	Neutral
The Scotsman	73,000	42.5	21.9	87.7	Neutral
Scotland on Sunday	72,000	51.4	18.1	84.7	Neutral
**Tabloid**					
Daily Record	484,000	48.3	69.4	89.1	Left
Sunday Mail	558,000	50.7	67.0	89.1	Left

*Not included in this analysis, as The Sun is not included in the Nexis database; included here for comparison.

For peer review only

Standards for Reporting Qualitative Research (SRQR)*

http://www.equator-network.org/reporting-guidelines/srqr/

Page/line no(s).

Title and abstract

**Title** - Concise description of the nature and topic of the study Identifying the study as qualitative or indicating the approach (e.g., ethnography, grounded theory) or data collection methods (e.g., interview, focus group) is recommended	Page 1, line 2
**Abstract** - Summary of key elements of the study using the abstract format of the intended publication; typically includes background, purpose, methods, results, and conclusions	Page 2, lines 18-48

Introduction

**Problem formulation** - Description and significance of the problem/phenomenon studied; review of relevant theory and empirical work; problem statement	Page 5-6, lines 60-101
**Purpose or research question** - Purpose of the study and specific objectives or questions	Page 6, line 102

Methods

**Qualitative approach and research paradigm** - Qualitative approach (e.g., ethnography, grounded theory, case study, phenomenology, narrative research) and guiding theory if appropriate; identifying the research paradigm (e.g., postpositivist, constructivist/ interpretivist) is also recommended; rationale**	Page 8, from line 131-148
**Researcher characteristics and reflexivity** - Researchers' characteristics that may influence the research, including personal attributes, qualifications/experience, relationship with participants, assumptions, and/or presuppositions; potential or actual interaction between researchers' characteristics and the research questions, approach, methods, results, and/or transferability	Page 8, lines 134-138
**Context** - Setting/site and salient contextual factors; rationale**	Page 7, lines 109-129 (as well as introduction, lines 60-101)
**Sampling strategy** - How and why research participants, documents, or events were selected; criteria for deciding when no further sampling was necessary (e.g., sampling saturation); rationale**	Page 7, lines 109-129,
**Ethical issues pertaining to human subjects** - Documentation of approval by an appropriate ethics review board and participant consent, or explanation for lack thereof; other confidentiality and data security issues	Page 19, line 362
**Data collection methods** - Types of data collected; details of data collection procedures including (as appropriate) start and stop dates of data collection and analysis, iterative process, triangulation of sources/methods, and modification of procedures in response to evolving study findings; rationale**	Page 7-9, lines 109-161

For peer review only - http://bmjopen.bmj.com/site/about/guidelines.xhtml

**Data collection instruments and technologies** - Description of instruments (e.g., interview guides, questionnaires) and devices (e.g., audio recorders) used for data collection; if/how the instrument(s) changed over the course of the study	Page 8, lines 131-148
**Units of study** - Number and relevant characteristics of participants, documents, or events included in the study; level of participation (could be reported in results)	Page 7, lines 119-129
**Data processing** - Methods for processing data prior to and during analysis, including transcription, data entry, data management and security, verification of data integrity, data coding, and anonymization/de-identification of excerpts	Page 9, lines 149 - 156
**Data analysis** - Process by which inferences, themes, etc., were identified and developed, including the researchers involved in data analysis; usually references a specific paradigm or approach; rationale**	Page 8, lines 131 - 141 and 149 - 156
**Techniques to enhance trustworthiness** - Techniques to enhance trustworthiness and credibility of data analysis (e.g., member checking, audit trail, triangulation); rationale**	Page 8, lines 142-148

Results/findings

**Synthesis and interpretation** - Main findings (e.g., interpretations, inferences, and themes); might include development of a theory or model, or integration with prior research or theory	Page 9-12, lines 169-138 (as well as discussion, page 13-15, lines 240-296)
**Links to empirical data** - Evidence (e.g., quotes, field notes, text excerpts, photographs) to substantiate analytic findings	Page 9-12, lines 169-138

Discussion

**Integration with prior work, implications, transferability, and contribution(s) to the field -** Short summary of main findings; explanation of how findings and conclusions connect to, support, elaborate on, or challenge conclusions of earlier scholarship; discussion of scope of application/generalizability; identification of unique contribution(s) to scholarship in a discipline or field	Page 13-17, lines 240-314
**Limitations** - Trustworthiness and limitations of findings	Page 16-17, lines 315 – 331

Other

**Conflicts of interest** - Potential sources of influence or perceived influence on study conduct and conclusions; how these were managed	Page 18, line 356-361
**Funding** - Sources of funding and other support; role of funders in data collection, interpretation, and reporting	Page 18, line 352-355

*The authors created the SRQR by searching the literature to identify guidelines, reporting standards, and critical appraisal criteria for qualitative research; reviewing the reference lists of retrieved sources; and contacting experts to gain feedback. The SRQR aims to improve the transparency of all aspects of qualitative research by providing clear standards for reporting qualitative research.

For peer review only

For peer review only - http://bmjopen.bmj.com/site/about/guidelines.xhtml

**The rationale should briefly discuss the justification for choosing that theory, approach, method, or technique rather than other options available, the assumptions and limitations implicit in those choices, and how those choices influence study conclusions and transferability. As appropriate, the rationale for several items might be discussed together.

Reference:
O'Brien BC, Harris IB, Beckman TJ, Reed DA, Cook DA. **Standards for reporting qualitative research: a synthesis of recommendations.** *Academic Medicine*, Vol. 89, No. 9 / Sept 2014 DOI: 10.1097/ACM.0000000000000388

For peer review only
